# NR4A Receptors Differentially Regulate NF-κB Signaling in Myeloid Cells

**DOI:** 10.3389/fimmu.2017.00007

**Published:** 2017-01-23

**Authors:** Caitriona McEvoy, Monica de Gaetano, Hugh E. Giffney, Bojlul Bahar, Eoin P. Cummins, Eoin P. Brennan, Mary Barry, Orina Belton, Catherine G. Godson, Evelyn P. Murphy, Daniel Crean

**Affiliations:** ^1^School of Medicine, University College Dublin, Dublin, Ireland; ^2^Conway Institute for Biomolecular and Biomedical Science, University College Dublin, Dublin, Ireland; ^3^Diabetes and Complications Research Centre, Conway Institute for Biomolecular and Biomedical Science, University College Dublin, Dublin, Ireland; ^4^School of Veterinary Medicine, University College Dublin, Dublin, Ireland; ^5^International Institute of Nutritional Sciences and Applied Food Safety Studies, University of Central Lancashire, Preston, UK; ^6^St. Vincent’s University Hospital, Dublin, Ireland

**Keywords:** Nr4a receptors, NF-κB, myeloid cells, CCL20, repression

## Abstract

Dysregulation of inflammatory responses is a hallmark of multiple diseases such as atherosclerosis and rheumatoid arthritis. As constitutively active transcription factors, NR4A nuclear receptors function to control the magnitude of inflammatory responses and in chronic inflammatory disease can be protective or pathogenic. Within this study, we demonstrate that TLR4 stimulation using the endotoxin lipopolysaccharide (LPS) rapidly enhances NR4A1–3 expression in human and murine, primary and immortalized myeloid cells with concomitant gene transcription and protein secretion of MIP-3α, a central chemokine implicated in numerous pathologies. Deficiency of NR4A2 and NR4A3 in human and murine myeloid cells reveals that both receptors function as positive regulators of enhanced MIP-3α expression. In contrast, within the same cell types and conditions, altered NR4A activity leads to suppression of LPS-induced MCP-1 gene and protein expression. An equivalent pattern of inflammatory gene regulation is replicated in TNFα-treated myeloid cells. We show that NF-κB is the critical regulator of NR4A1–3, MIP-3α, and MCP-1 during TLR4 stimulation in myeloid cells and highlight a parallel mechanism whereby NR4A activity can repress or enhance NF-κB target gene expression simultaneously. Mechanistic insight reveals that NR4A2 does not require DNA-binding capacity in order to enhance or repress NF-κB target gene expression simultaneously and establishes a role for NF-κB family member Relb as a novel NR4A target gene involved in the positive regulation of MIP-3α. Thus, our data reveal a dynamic role for NR4A receptors concurrently enhancing and repressing NF-κB activity in myeloid cells leading to altered transcription of key inflammatory mediators.

## Introduction

The NR4A family of nuclear receptors control inflammatory processes and function as important transcriptional regulators in several disease states characterized by aberrant inflammation including atherosclerosis and arthritis (1–3). The NR4A family comprises three members, namely, NR4A1 (Nur77), NR4A2 (Nurr1), and NR4A3 (Nor-1). As orphan receptors, they have no known endogenous ligand(s) and are regulated primarily at the level of gene transcription, posttranslational modifications, and protein–protein interactions (1–3). However, these receptors can be activated by select exogenous agents such as the photochemical-based compound 1,1-bis (3′-indolyl)-1-(*p*-chlorophenyl) methane (C-DIM12), an NR4A2 activator, and the fungal metabolite Cytosporone-B (Csn-B), an NR4A1 agonist (4–6). These receptors control and limit inflammatory responses primarily by acting as feedback regulators of NF-κB signaling, and more recently have been identified as promoters of inflammatory resolution processes (1–3, 7). Thus, pharmacological modulation of NR4A receptors has wide-ranging therapeutic potential to control hyperinflammatory responses associated with inflammatory diseases (1–3, 8).

NR4A receptors have profound effects on NF-κB, paradoxically repressing or enhancing its activity depending on tissue, cell type, or disease context (9–14). NR4A1 and 2 depletion promotes polarization of macrophages toward a pro-inflammatory phenotype during TLR4 or TNFα stimulation, and this is associated with enhanced NF-κB activity (9–14). NF-κB enhancement is demonstrated through several mechanisms: increased nuclear translocation, phosphorylation, and DNA binding by NF-κB/p65 leading to increased expression of key inflammatory genes including IL-12, iNOS, TNFα, IL-8, IL-6, and MCP-1 (9–14). Recently, it has been shown that NR4A3 depletion in myeloid cells parallels the responses observed by other family members during TLR4 stimulation, displaying enhanced expression of NF-κB-driven TNFα and MCP-1 (14). In contrast to these observations, Pei et al. revealed that ectopic overexpression of NR4A1–3 in murine macrophage cells enhances lipopolysaccharide (LPS)-induced promoter expression of the pro-inflammatory mediator IKKi (15). Furthermore, in rheumatoid arthritis synoviocyte cells, NR4A2 cooperates with NF-κB/p65 to promote IL-8 production during TNFα receptor stimulation (10). Hence, the ability of NR4A1–3 receptors to repress or enhance NF-κB target gene expression highlights a dynamic relationship between these nuclear factors and the need for appropriate models when investigating the impact of NR4A activity on inflammatory outcome.

In this study, we focus on the role NR4A2 and NR4A3 receptors play in regulating key inflammatory target gene expression and NF-κB activity in myeloid cells. TLR4 stimulation using the endotoxin LPS drives rapid expression of NR4A1–3 gene and protein expression in human and murine, primary and immortalized myeloid cells with concomitant expression of MIP-3α mRNA and protein release. Deficiency of NR4A2 and 3 reveals a novel role for NR4A receptors as important positive regulators of inflammatory driven MIP-3α in both human and murine myeloid cells. Within the same cell conditions, we reveal that NR4A2 and 3 function simultaneously as repressors of inflammatory-driven MCP-1. Furthermore, we demonstrate that NF-κB is the central transcriptional regulator of NR4As, MIP-3α, and MCP-1 expression within TLR4-stimulated myeloid cells, confirming that depletion of NR4A receptor activity is capable of concurrent repression and enhancement of NF-κB target gene expression. NR4A2 does not require the ability to bind DNA in order to enhance or repress NF-κB target gene expression. Further investigations using mouse embryonic fibroblast (MEF) cells lacking p65, p50, p52, or Relb reveal that Relb is required to mediate MIP-3α expression. Furthermore, we establish NF-κB/p65 as a critical regulator of Relb during TLR4-driven responses. Our data highlight NR4A2 and 3 as important receptors in the regulation of Relb in myeloid cells during TLR4 stimulation. This positive regulation of Relb by the NR4A receptors, coupled with the involvement of Relb in the regulation of MIP-3α, identifies a crucial mechanism in the dual activation/repression of NF-κB/NR4A target gene expression. Additionally, *in vivo* analysis demonstrates a significant increase in NR4A3 and MIP-3α expression specifically in internal carotid (IC) plaques from symptomatic patients. Subsequent PCR array analysis reveals that NR4A2 and 3 are key regulators of novel downstream target connective tissue growth factor (CTGF), an important atherogenic and fibrotic factor. Consistent with above mentioned target genes, we reveal that NF-κB, upstream of NR4A receptor activation, is the critical regulator of inflammatory-driven CTGF. Thus, we demonstrate the critical role NR4A genes play in modulating appropriate NF-κB target gene expression during inflammatory activation in myeloid cells.

## Materials and Methods

### Cell Culture and Treatments

Human monocytic THP-1 and murine macrophage RAW 264.7 cells obtained from American type culture collection (ATCC^®^ TIB-202™ and TIB-71™, respectively) were cultured in RPMI-1640—GlutaMax™ media (Life Technologies™) and DMEM 6546 (Sigma-Aldrich^®^), respectively. MEF cells lacking NF-κB subunit p65 (p65^−/−^), p50 (p50^−/−^), p52 (p52^−/−^), Relb (Relb^-/−^), and wild-type (WT) controls were a generous gift from Alexander Hoffman (UCLA) and cultured in DMEM 6546 (Sigma-Aldrich^®^). All cell media were supplemented with 10% FBS, 100 U/ml penicillin, and 100 µg/ml streptomycin. Cells were cultured at 21% O_2_, 5% CO_2_, and maintained in a humidified tissue culture incubator at 37°C balanced using nitrogen. Stable knockdown cells were maintained in an additional 5 µg/ml Puromycin dihydrochloride (Sigma-Aldrich^®^) for stable knockdown selectivity. Reagents such as LPS, BAY-11-7082, and Cytosporone-b (Csn-B) were supplied by Sigma-Aldrich, and TNFα was supplied by RnD systems.

### Primary Cell Isolation

Peripheral venous blood was collected from healthy volunteers at the Conway Institute, University College Dublin (UCD). Institutional review board approval was obtained from the Ethics Committee at St. Vincent’s University Hospital (UCD affiliated teaching hospital), and written informed consent was obtained from all volunteers. A total of 30 ml of blood was collected into a syringe, layered slowly onto polymorphoprep solution (1:1), and centrifuged at 500 × *g* for 35 min at 20°C with the brake off. The mononuclear layer was removed and mixed with equal volumes of 0.45% NaCl by gentle inversion followed by centrifugation at 400 × *g* for 10 min at 20°C with the brake on. Supernatant was discarded and pellet was re-suspended in 12 ml ice-cold water and mixed by inverting gently for 1 min followed by the addition of 12 ml 1.8% NaCl and subsequent centrifugation at 300 × *g* for 5 min at 20°C with the brake on. Cells were then re-suspended in media, counted, and seeded at a density of 2.5 × 10^5^ cells/ml for RNA experiments.

### Patient Recruitment and Definition

The study was approved by the Ethics Committee of St. Vincent’s University Hospital, Dublin, and in accordance with International guidelines and Helsinki declaration principles. All patients (*n* = 6) gave written informed consent. This patient cohort has been recently described and analyzed (16). Patients, symptomatic and asymptomatic, with clinical and angiographic evidence of atherosclerosis undergoing revascularization surgery were recruited to the study, and clinical details obtained for patients are supplied as Table S1 in Supplementary Material. Three “symptomatic” patients [defined as patients who had had a first-ever transient ischemic attack or non-disabling ischemic stroke from atherosclerotic stenosis and without any known history or diagnosis of cardiovascular disease (CVD)]; and three “asymptomatic” patients (defined as those without any previous ischemic events or known CVD) were studied.

### Tissue Specimen Preparation

Immediately following carotid endarterectomy, human carotid atherosclerotic plaque specimens were placed in RNAlater^®^ solution (Thermo Fisher), and stored at −80°C. Each plaque specimen was then dissected into four distinct sections: common carotid (CC), Internal Carotid (IC), external carotid (EC), and relatively disease free (RDF), as shown in the schematic (Figure S3A in Supplementary Material). Subsequent analysis was performed on IC and RDF samples only as described below in qRT-PCR methods.

### Stable Lentiviral Knockdown

Stable knockdown of human NR4A2, NR4A3, and murine NR4A2 was achieved with the transduction of shRNA using a Mission™ Lentiviral packaging mix as per instructions (Sigma-Aldrich) using THP-1. For controls, cells were transduced with scrambled shRNA (Sigma-Aldrich). Briefly, for THP-1 cells, 150 µl of cells from a stock concentration of 2.5 × 10^5^ cells/ml were pipetted into a 96-well plate; for Raw mac 264.7 cells, 250 µl of cells from a stock concentration of 1.5 × 10^5^ cells/ml were pipetted into a 24-well plate and both were incubated overnight. Hexadimethrine bromide was added to a final concentration of 8 µg/ml followed by the addition of 10 µl of lentiviral particles and subsequent incubation for 48 h in a tissue culture incubator at 37°C. Subsequent to this, puromycin was added to the cells (5 µg/ml), and medium was changed every 3–4 days until resistant colonies were identified and subsequently expanded. Western blotting was used to confirm knockdown levels for human THP-1 cells and qRT-PCR was used for murine Raw mac 264.7.

### Transient Transfections

Mouse embryonic fibroblast cells were transfected with previously described/published plasmids p-CMX-NR4A2 (WT) and p-CMX-NR4A2 mutant (C283G) (10, 17). MEF cells were trypsinized, spun down to obtain pelleted cells, re-suspended in fresh media, and brought to a final cell concentration of 1.2 × 10 cells/ml. Optimem, plasmid, and transfection reagent (TransIT2020) (Mirus) were mixed and left for 30 min to allow complexes to form as per manufacturer’s instructions in the ratio 100 μl:500 ng:3 μl (Optimem:Plasmid:TransIT2020). Subsequently 100 µl of mixture/complexes was added to 2 ml of cell suspension in 6-well plates and incubated for 48 h. At 48 h, cells were treated as described in figure legends.

### Western Blotting

Whole-cell lysates: suspension cells (THP-1) were pelleted by centrifugation @ 1,200 RPM followed by re-suspension into 100 µl radio-immuno-precipitation assay (RIPA) buffer. The re-suspended pellets were then pipetted back onto their respective treatment dishes and were scraped into a new Eppendorf tube to ensure collection of floating cells and cells that adhered during the course of stimulations. Adherent cells (Raw mac 264.7) and MEFs were scraped directly into RIPA buffer. Cell suspensions were then agitated vigorously on a rocker for 30 min at 4°C followed by centrifugation at 15,000 × *g* for 15 min. Supernatants were removed as whole-cell extracts and stored at −20°C.

Protein content was assessed by the Bradford method (Bio-Rad Laboratories) and electrophoresed on 10% SDS PAGE gel followed by transfer to nitrocellulose membranes (Bio-Rad). Protein expression levels were measured by Western blot analysis using specific antibodies for NR4A2 and NR4A3 (RnD systems); p65 (Cell Signaling); Relb (Cell Signaling); CTGF (Santa Cruz); and β-tubulin (Sigma). Briefly, nitrocellulose membranes were incubated with primary antibodies overnight at 4°C followed by washing three times for 10 min with 1× TBST [20 mM Tris, 137 mM NaCl, and 0.05% (v/v) tween 20] and subsequent incubation for 1 h with species-specific HRP-conjugated secondary antibodies at room temperature. Membranes were washed again as described followed by signal detection using enhanced chemiluminescence reagent (Pierce). Densitometric analysis included for Western blot data was determined using LI-COR^®^ Image Studio Lite version 3.1. Briefly, the intensity of proteins of interest (NR4A2, NR4A3, p65, Relb, and CTGF) was quantified using LI-COR program relative to loading control protein (β-tubulin).

### ELISA

Medium from THP-1-treated cells was centrifuged briefly to remove cell debris, and supernatant was stored at −20°C. MCP-1 and MIP-3α (RayBiotech) protein levels were measured using an enzyme-linked immunosorbent assay. IFNγ, IL-12p70, IL-10, IL-1β, IL-6, IL-8, and TNFα were measured using the Human ProInflammatory 7-Plex Tissue Culture Kit (MSC). ELISA was performed as per manufacturer’s instructions. Changes incorporated into the protocols specific for our data are as follows. For each ELISA, the dilution value was calculated to yield optimum results within the linear range of the assay. From this, we determined that samples were used neat on the 7-Plex ELISA, diluted 1:10 for MCP-1 and 1:6 for MIP-3α.

### qReal-Time PCR (qRT-PCR)

RNA was extracted from *in vitro* cells using the column-based EZNA^®^ total RNA extraction kit (Omega Bio-Tek, GA, USA). Trizol (Invitrogen) extraction was used to isolate total RNA from patient samples stored in RNAlater^®^. cDNA synthesis was subsequently performed as previously reported (14). qRT-PCR was performed using SYBR Green Master Mix (Applied Biosystems) and on an ABI 7300 thermocycler (Applied Biosystems). Primer pair sequences used are detailed in Table S2 in Supplementary Material. Relative expression/abundance levels of target gene transcripts were determined using qBase plus software (Biogazelle, Ghent University, Belgium) with GAPDH or β-actin (*in vitro* samples) or ribosomal 18s (patient samples) as a reference target. Results are expressed as fold over untreated control (FOC).

### PCR Array

An in-house PCR array, consisting of 92 inflammatory genes and 4 reference genes, was performed. For this array, cDNA synthesis was performed using 500 ng of total RNA using the RT^2^ First Strand Kit (Qiagen Ltd.) according to the manufacturer’s instructions. Real-time reverse transcriptase PCR was performed on a 20 µl reaction mixture per well containing 1 µl cDNA (after 1:5 dilution), 9 µl water, and 10 ml RT^2^ SYBR Green/ROX qPCR master mix (SABiosciences) in a 7300 Real-time PCR system (Applied Biosystems). Using a web-based PCR array data analysis tool (SABiosciences), the *C*_T_ values of the target genes were normalized against the four reference genes (GAPDH, HPRT1, TBP, and ACTB) and the relative abundance of gene transcripts were calculated as ${\text{log}}_{\;}^{2△C_{\text{T}}}$. Subsequently, expression fold changes were calculated as fold over untreated control sample (FOC) and are displayed in Table S3 in Supplementary Material, alongside raw *C*_T_ values. Using the PCR array data tool, a clustergram was made and displayed in Figure 6A. A clustergram performs non-supervised hierarchical clustering of the entire data set and is represented as a heat map with a dendrogram showing individual genes and overall treatment groups with a similar regulation pattern.

### Promoter Studies and Analysis

The Genomatix software suite program MatInspector (© Genomatix Software GmbH) was used to analyze the human and murine MIP-3α promoter 500 bp upstream of the transcription start site (TSS). Two stringency tests were applied as per Genomatix software instructions in order to select binding sites that displayed the best probability as “real” binding motifs for transcription factors (Core similarity of 1.0 and matrix similarity >0.90). The first test is the core similarity test and the maximum core similarity of 1.0 is only reached when the highest conserved bases of a matrix match exactly in the given sequence. The second test is the matrix similarity test and the score must be greater than 0.90.

### Statistical Analysis

All data is presented as mean ± SEM and representative Western blots for a minimum of three individual *n* numbers (unless otherwise stated). Statistical significance was performed using a two-tailed Student’s *t*-test for patient tissue analysis and one-way analysis of variance followed by Tukey’s *post hoc* test was utilized throughout all other results (GraphPad Prism^®^).

## Results

### TLR4 Stimulation Drives NR4A Gene and Protein Expression in Myeloid Cells

TLR4 ligands, including LPS, have been shown to drive NR4A gene expression in immune cells (14, 18). Several studies have shown in both human and mouse macrophages, using NR4A1–3 overexpression and/or knockout cells, that NR4A receptors regulate expression of several downstream NF-κB target genes including IKKi, TNFα, MCP-1, iNos, IP-10, and IL-12 (9, 11, 13–15). NR4A family members can modulate NF-κB activity downstream of TLR4 signaling in a dynamic fashion, either repressing or enhancing NF-κB target gene expression (9, 11, 13–15). The majority of the work to date suggests that NR4A1 and 2 depletion promotes a pro-inflammatory macrophage phenotype, displaying enhanced NF-κB activity (9, 11, 13–15). However, there are minimal studies elucidating the role monocyte/macrophage NR4A3 plays in modulating such regulatory pathways.

**Figure 1:**
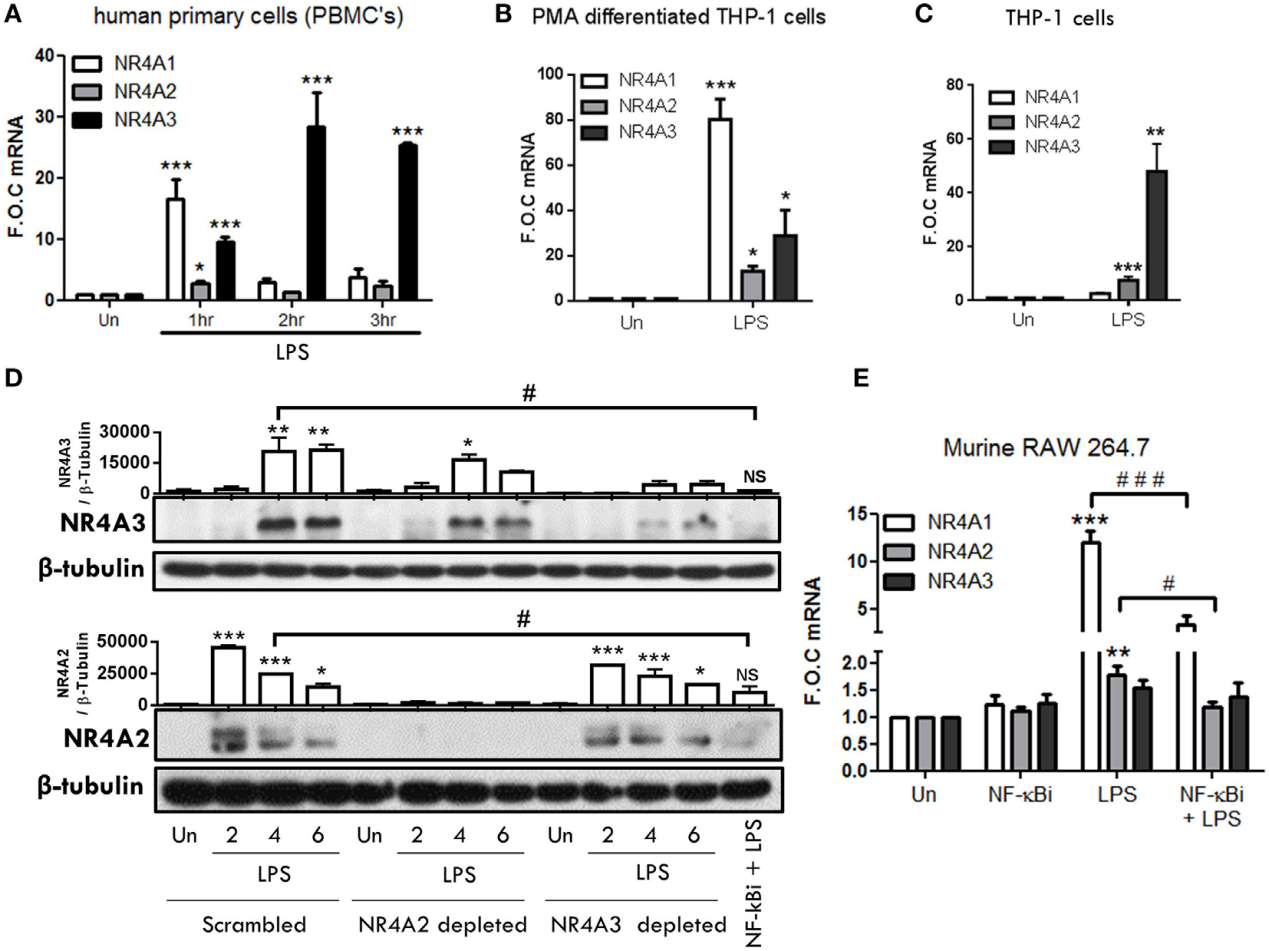
**TLR4 stimulation drives NR4A genes in monocyte/macrophage cells**. **(A)** Human primary PBMCs were exposed to 1 µg/ml lipopolysaccharide (LPS) for 1, 2, and 3 h. **(B)** THP-1 cells differentiated using 20 ng/ml PMA were exposed to 1 µg/ml LPS for 2 h. **(C)** Undifferentiated THP-1 cells were exposed to 100 ng/ml LPS for 2 h. **(D)** Undifferentiated THP-1 cells transduced with shRNA directed against scrambled non-target control, NR4A2, or NR4A3 were treated with 1 µg/ml LPS for 0, 2, 4, and 6 h. Additionally, scrambled non-target control cells were pretreated with NF-κB inhibitor BAY-11-7082 (NF-κBi) (10 µM) for 1 h followed by 4 h 1 µg/ml LPS. Whole-cell lysates were then prepared from all treatments. **(E)** Murine raw mac 264.7 cells were pretreated with (NF-κBi) (10 µM) for 1 h followed by exposure to 1 µg/ml LPS for 1 h. Analysis: RNA was isolated at indicated time and RT-PCR was performed to assess levels of NR4A1, 2, 3, and control gene GAPDH **(A–C,E)**. Western blot analysis was performed on whole-cell lysates for NR4A2, NR4A3, and loading control β-Tubulin **(D)**. Densitometric analysis included for Western blot data was determined using LI-COR^®^ Image Studio Lite version 3.1, and band density of target proteins (NR4A2 and NR4A3) normalized to loading control (β-Tubulin) are displayed above relevant treatments (*n* = 2). Data are expressed as fold over untreated control (FOC) ± SEM for *n* = minimum of three individual experiments or representative Western blot for *n* = minimum of two individual experiments. *p < 0.05, **p < 0.01, ***p < 0.001 treatments compared to untreated control (Un).

To examine TLR4-driven NR4A2 and NR4A3 gene and protein expression, we utilized a range of human myeloid cells including primary human mononuclear cells (PBMCs), THP-1s, Phororbol-12-myristate-13-acetate (PMA) differentiated THP-1s, and murine RAW mac264.7 cells. In human PBMCs, we reveal temporal changes in NR4A1–3 gene expression in response to LPS (1.0 µg/ml) (Figure 1A). We observe NR4A1 gene expression rises sharply and significantly at 1 h and is reduced by 2 and 3 h. NR4A2 gene expression is significantly increased at 1 h albeit to a very low level, while NR4A3 gene expression is significantly increased at 1, 2, and 3 h post stimulation (Figure 1A). Examining PMA (20 nM) differentiated THP-1 cells reveals a significant induction of all NR4A family members at 2 h exposed to 1.0 µg/ml LPS (Figure 1B). Using undifferentiated THP-1 cells, we observe a significant and selective induction of NR4A2 and 3 gene expression within 2 h LPS (100 ng/ml) treatment (Figure 1C) with minimal changes in NR4A1 expression.

Thus, we examined temporal levels of NR4A2 and 3 protein expression in undifferentiated THP-1 cells exposed to LPS (1.0 μg/ml) (Figure 1D). Undifferentiated THP-1 cells were chosen for extended analysis based on their similarity to the expression profile observed in primary PBMCs for NR4A receptors in response to LPS. Additionally, we have previously shown undifferentiated THP-1 cells closely reflect primary cell responses to various stimuli such as LPS and adenosine and support that they are a relevant immortalized cell line to use in such myeloid cell studies (14). NR4A3 protein is dramatically increased within 4 h and maintained at 6 h post LPS, while NR4A2 is enhanced as early as 2 h with protein levels reducing over 4–6 h (Figure 1D) revealing distinct temporal changes in protein expression under these cellular conditions. We and others have shown previously that LPS-driven NR4A transcription is NF-κB dependent (14, 18). Here, we support the studies confirming that pretreatment with the NF-κB inhibitor BAY 11-7082 (10 µM) abolishes LPS-driven NR4A2 and 3 protein expression (Figure 1D). We next examined the expression of NR4A genes in response to LPS in the murine macrophage cell line Raw Mac 264.7 (Figure 1E). LPS (1 µg/ml) significantly drives NR4A1 and NR4A2 gene expression within 1 h; in contrast, NR4A3 mRNA levels remain unchanged (Figure 1E). Using the NF-κB inhibitor BAY 11-7082 (NF-κBi) (10 µM), we further establish that LPS-induced NR4A1/2 is NF-κB dependent in these cells (Figure 1E).

NR4A2 and 3 virally delivered shRNA depleted cells were utilized (14) to further identify the transcriptional role of NR4A2 and 3 in TLR4 driven gene expression. As shown in Figure 1D, which incorporates NR4A2/3 depleted cells alongside scrambled control cells, we demonstrate a significant depletion of NR4A2 (≈96%) and NR4A3 (≈79%) (% calculated from densitometry band intensity values) (Figure 1D). Taken together, we establish that LPS drives temporal expression of NR4A1–3 genes in human and murine myeloid cells and further demonstrate that LPS-driven NR4A2 and 3 expression is NF-κB dependent.

### NR4A Receptors Negatively and Positively Regulate NF-κB Target Gene Expression Simultaneously

**Figure 2:**
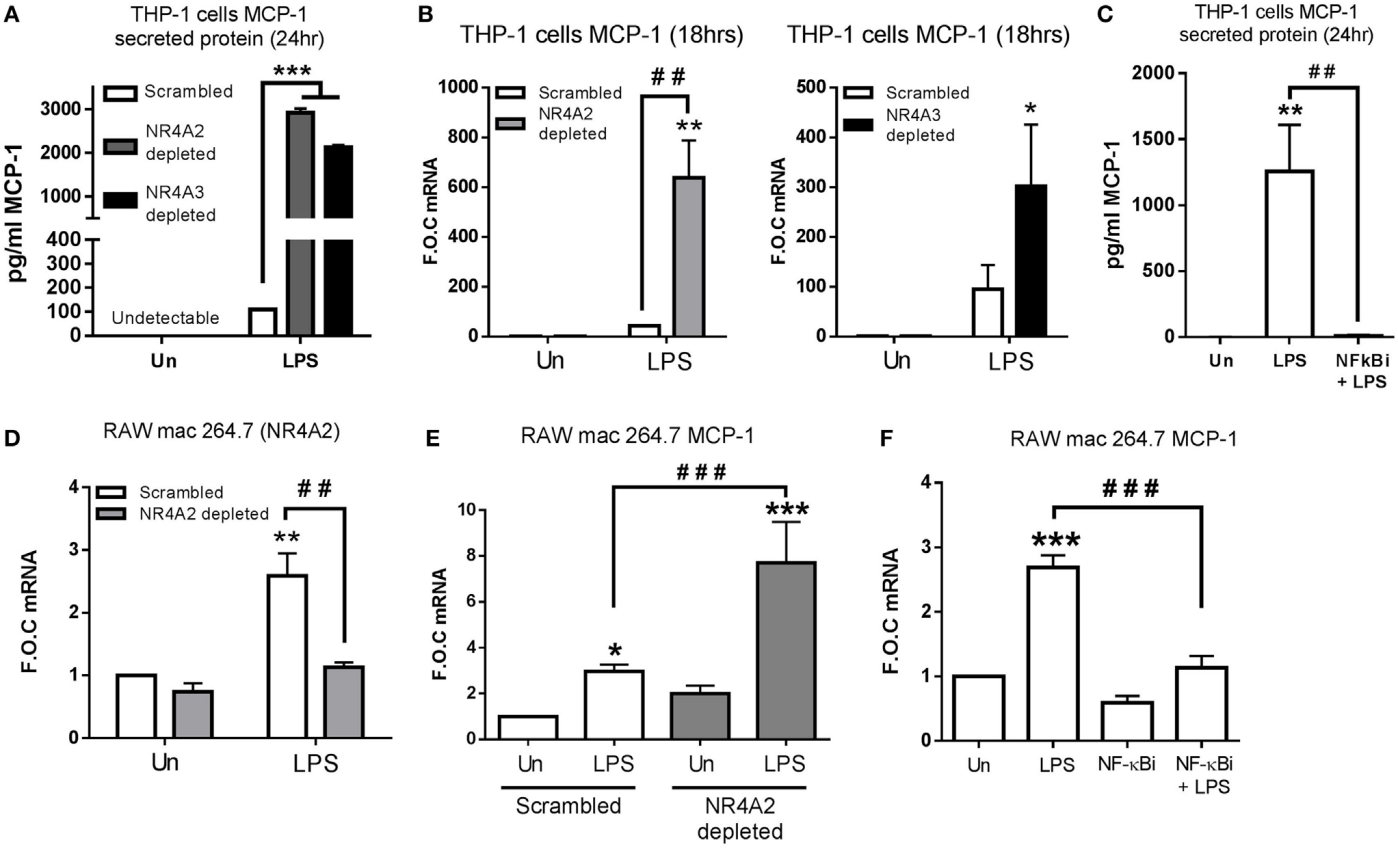
**NR4A2 and 3 negatively regulate TLR4 driven MCP-1 in human and murine myeloid cells**. **(A,B)** Undifferentiated THP-1 cells transduced with shRNA directed against scrambled non-target control, NR4A2, or NR4A3 were treated with 1 µg/ml lipopolysaccharide (LPS) for 18 h **(B)** and 24 h **(A)**, followed by media collection and RNA isolation, respectively. **(C)** Undifferentiated THP-1 cells were pretreated with 10 µM NF-κB inhibitor (NF-κBi) for 1 h followed by the addition of 1 µg/ml LPS for 24 h, followed by media collection. **(D)** Murine raw mac 264.7 cells transduced with shRNA directed against scrambled non-target control and NR4A2 were exposed to LPS for 1 h. **(E)** Murine raw mac 264.7 cells transduced with shRNA directed against scrambled non-target control and NR4A2 were exposed to 1 µg/ml LPS for 2 h. **(F)** Murine raw mac 264.7 cells were pretreated with 10 µM NF-κB inhibitor (NF-κBi) for 1 h followed by exposure to 1 µg/ml LPS for 2 h. Analysis: ELISA analysis was performed for MCP-1 protein detection on media collected at times indicated **(A,C)**. RNA was isolated and RT-PCR was performed at indicated times to assess levels of MCP-1 NR4A2 and control gene GAPDH **(B,D–F)**. Un, untreated control. Data are expressed as fold over untreated control (FOC) or pg/ml ± SEM for *n* = minimum of three individual experiments. *p < 0.05, **p < 0.01, ***p < 0.001 treatments compared to untreated control (Un). ^###^*p* < 0.01, ^###^*p* < 0.001 treatments compared displayed here using a bar attachment.

NR4A receptors have the ability to promote or repress NF-κB target gene expression, albeit to date such divergent mechanisms have been established distinctly, and not simultaneously. We therefore investigated the effect of NR4A depletion on a number of known NF-κB target genes in myeloid cells. A multiple ELISA was performed to quantify IFNγ, IL-12p70, IL-10, IL-1β, IL-6, IL-8, TNFα, and additional single ELISA for MCP-1 and MIP-3α expression levels. These targets were selected having identified them as NF-κB-regulated genes using the NF-κB inhibitor BAY-11-7082 in THP-1 cells [data not shown, Figure 2 and Ref. (9, 14)]. Second, NR4A receptors have been shown to be critical in regulating their appropriate expression during inflammatory responses by our group and others (3, 9, 10, 14, 19).

ELISA analysis reveals NR4A2 and 3 depletion results in a more pronounced TLR4 response, displaying enhanced LPS-driven IL-12p70, Il-1β, IL-10, TNF-α, and IL-6 protein secretion (Figure S1 in Supplementary Material). We also observed enhanced protein secretion and mRNA expression of MCP-1 within NR4A2 and 3 depleted cells, which was more profound than any of the other targets described above (Figures 2A,B). Furthermore, we show that this LPS-induced MCP-1 is inhibited by the use of the NF-κB inhibitor BAY-11-7082 (NF-κBi), confirming MCP-1 to be an NF-κB-dependent gene (Figure 2C). As NR4A2 and NR4A3 display distinct expression changes in murine Raw mac 264.7 cells (Figure 1E), stably depleted NR4A2 cell line was selected using viral delivery of shRNA directed against NR4A2 and scrambled control. Gene expression analysis reveals that 95% depletion of LPS (1 µg/ml) induced NR4A2 gene expression in NR4A2 shRNA-transduced cells compared to scrambled control (Figure 2D). We further reveal scrambled Raw mac 264.7 cells exposed to LPS for 1 h results in significant MCP-1 gene expression, and this regulation is further potentiated with NR4A2 depletion (Figure 2E). Additionally, using the NF-κB inhibitor BAY 11-7082 (NF-κBi), we show that LPS-driven MCP-1 in Raw mac 264.7 cells is also NF-κB dependent (Figure 2F).

**Figure 3:**
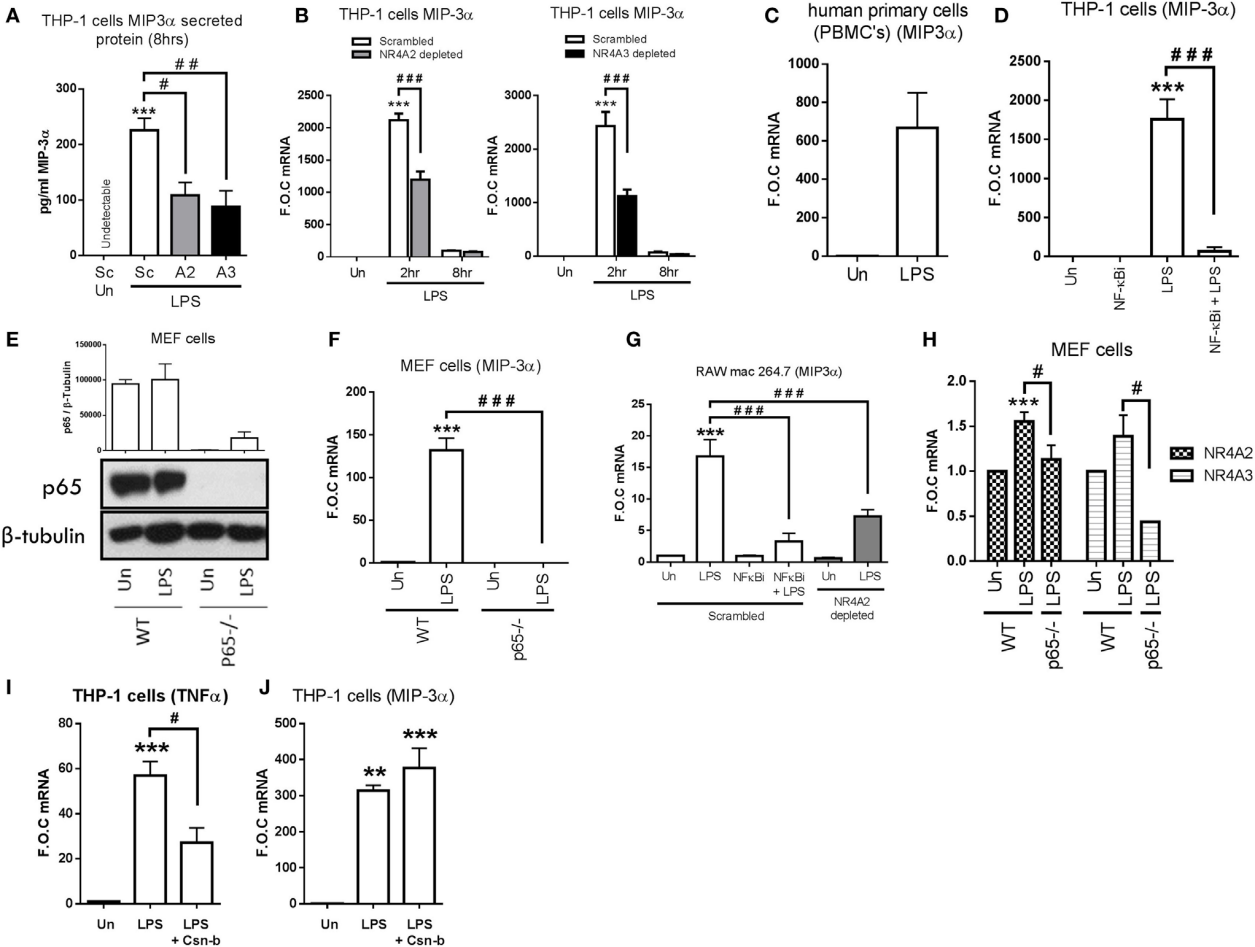
**NR4As positively regulate TLR4 driven MIP-3α in human and murine monocyte/macrophage cells**. **(A)** Undifferentiated THP-1 cells transduced with shRNA directed against scrambled non-target control (Sc), NR4A2 (A2), or NR4A3 (A3) were treated with 1 µg/ml lipopolysaccharide (LPS) for 8 h, followed by media collection. **(B)** Undifferentiated THP-1 cells transduced with shRNA directed against scrambled non-target control, NR4A2, or NR4A3 were treated with 1 µg/ml LPS for 0, 2, and 8 h. **(C)** Human primary PBMCs were exposed to 1 µg/ml LPS for 2 h. **(D)** Undifferentiated THP-1 cells were pretreated with 10 µM NF-κB inhibitor (NF-κBi) for 1 h followed by the addition of 1 µg/ml LPS for 2 h. **(E,F)** Mouse embryonic fibroblast (MEF) cells lacking p65^−/−^ and wild-type (WT) controls were exposed to 1 µg/ml LPS for 4 h **(E)** and 2 h **(F)**. **(G)** Murine raw mac 264.7 cells transduced with shRNA directed against scrambled non-target control (pretreated with 10 µM NF-κB inhibitor (NF-κBi) for 1 h) and NR4A2 were subsequently treated with of 1 µg/ml LPS for 1 h. **(H)** MEF cells lacking p65^−/−^ and WT controls were exposed to 1 µg/ml LPS for 2 h. **(I,J)** Undifferentiated THP-1 cells were pretreated with 100 nM Cytosporone-B (Csn-b) for 30 min followed by the addition of 100 ng/ml LPS for a further 1.5 h. Analysis: ELISA was performed at time indicated for MIP-3α protein **(A)**. RNA was isolated and RT-PCR was performed at indicated times to assess levels of MIP-3α, NR4A2, NR4A3, TNFα, and control gene GAPDH **(B–D,F,H,I,J)**. Whole-cell lysates were prepared at indicated time followed by Western blot analysis performed for both p65 and loading control β-tubulin **(E)**. Densitometric analysis included for Western blot data was determined using LI-COR^®^ Image Studio Lite version 3.1 and band density of target protein (p65) normalized to loading control (β-Tubulin) are displayed above relevant treatments. Un, untreated control. Data are expressed as fold over untreated control (FOC) or pg/ml ± SEM for *n* = minimum of three individual experiments. **p < 0.01, ***p < 0.001 treatments compared to untreated control (Un). ^#^*p* < 0.05, ^##^*p* < 0.01, ^###^*p* < 0.001 treatments compared displayed here using a bar attachment.

Intriguingly however, having demonstrated that NR4As negatively regulate the above NF-κB target genes, we observe LPS-dependent secretion of MIP-3α protein is significantly attenuated in NR4A2 and NR4A3 depleted cells (*p* ≤ 0.05 and *p* ≤ 0.01, respectively) (Figure 3A). Further analysis shows LPS drives MIP-3α gene expression within 2 h and expression returns to basal levels by 8 h. However, this induction is significantly attenuated (≈50%) in cells depleted of NR4A2 or NR4A3 expression (Figure 3B). Additionally, we observe a significant induction in MIP-3α gene expression in human PBMCs treated with 1 µg/ml LPS for 2 h (Figure 3C). Furthermore, LPS significantly drives MIP-3α mRNA in undifferentiated THP-1 cells and use of the NF-κB inhibitor, BAY 11-7082 (NF-κBi) (10 µM), confirms this induction is NF-κB dependent (Figure 3D). To further support NF-κB-dependent regulation of MIP-3α, we utilized mouse embryonic fibroblast (MEF) cells lacking the NF-κB subunit p65 (p65^−/−^) and WT control (Figure 3E). We demonstrate that LPS drives MIP-3α gene expression in WT MEF cells and that this induction is abolished in the p65^−/−^ MEF cells (Figure 3F). Raw mac 264.7 cells exposed to 1 µg/ml LPS for 1 h results in a significant induction of MIP-3α gene expression and that this induction is significantly attenuated in NR4A2-depleted cells (Figure 3G). Using the NF-κB inhibitor BAY 11-7082 (NF-κBi) (10 µM), LPS-driven MIP-3α is shown to be NF-κB dependent in Raw mac 264.7 cells (Figure 3G). Importantly, using the MEF cells lacking the NF-κB subunit p65 (p65^−/−^) analysis confirms a significant reduction in LPS-driven MIP-3α gene expression compared to WT control (Figure 3F), and here we reveal that NR4A2 and NR4A3 expression is also reduced in the MEF p65^−/−^ cells during TLR4 stimulation using LPS compared to WT cells (Figure 3H).

In order to address the role of NR4A1 in mediating MIP-3α expression, we utilized the NR4A1 agonist Cytosporone-B (Csn-B). We confirm Csn-B activity within our cell system by examining the expression of TNFα, previously shown to be repressed by NR4A1 activity in myeloid cells (20). Here, we confirm that Csn-B co-treatment significantly attenuates LPS-driven TNFα (Figure 3I). Further analysis reveals that stimulation of THP-1 cells with LPS (100 ng/ml), in the presence of Csn-B, does not significantly alter MIP-3α induction (Figure 3J).

We next investigated whether this differential regulation of NF-κB targets by NR4A receptors was specific to LPS-TLR4-driven inflammatory responses by using TNFα in the above systems. Here, we reveal that TNFα drives MIP-3α expression and protein secretion in primary and THP-1 cells (Figures S2A,B in Supplementary Material). Additionally, we show that TNFα-driven MIP-3α is NF-κB dependent using the BAY 11-7082 NF-κB inhibitor (Figure S2C in Supplementary Material). We observe analogous regulation of MIP-3α expression in both scrambled control and NR4A2/3 depleted cells, demonstrating that this regulatory mechanism is not limited to TLR4 signaling (Figure S2D in Supplementary Material). Furthermore, we observe a similar pattern of regulation of MCP-1 in scrambled and NR4A2/3 depleted cells in response to TNFα (Figures S2E,F in Supplementary Material).

We have established using myeloid cells the involvement of NR4A2/3 receptors in the regulation of MIP-3α downstream of TLR4 or TNFα signaling. To expand these observations, we sought to examine the expression profile of NR4A2/3 and MIP-3α expression *in vivo* in human carotid plaque tissue, given the fact that both genes have been identified as important factors in the pathogenesis of atherosclerosis (21–25). Figure S3A in Supplementary Material identifies the specific regions of the carotid where portions examined are taken from, IC or RDF from asymptomatic and symptomatic patients, as described within the Section “Materials and Methods.” Our analysis reveals that, in symptomatic plaques, NR4A3 and MIP-3α expression is significantly elevated in the IC-diseased portion compared to their respective RDF or IC sections from asymptomatic patients. Interestingly, NR4A2 expression is not significantly increased in symptomatic patients compared to asymptomatic tissue (Figure S3B in Supplementary Material). Therefore, NR4A2 and NR4A3 *in vitro* have been shown to regulate MIP-3α gene expression, while *in vivo*, specific NR4A3 expression levels parallel MIP-3α expression suggesting NR4A3 may be involved in an *in vivo* setting, albeit patient numbers are limited and the role of NR4A3 regulating MIP-3α in such disease warrants further investigation.

Thus, data herein strengthen the hypothesis that NR4As are not merely repressors or enhancers of NF-κB target genes in isolation but can lead to distinct cellular outcomes by both positive and negative modulation of target gene expression concurrently. Thus, NR4A2 and 3 are key regulators of MIP-3α gene expression in human and murine myeloid cells. We demonstrate that NR4A2 and 3 receptors concurrently enhance and repress NF-κB-dependent target gene expression, positively regulating MIP-3α while negatively regulating targets such as MCP-1.

### NR4A2 Does Not Require DNA-Binding Capacity to Differentially Regulate NF-κB Target Gene Expression

**Figure 4:**
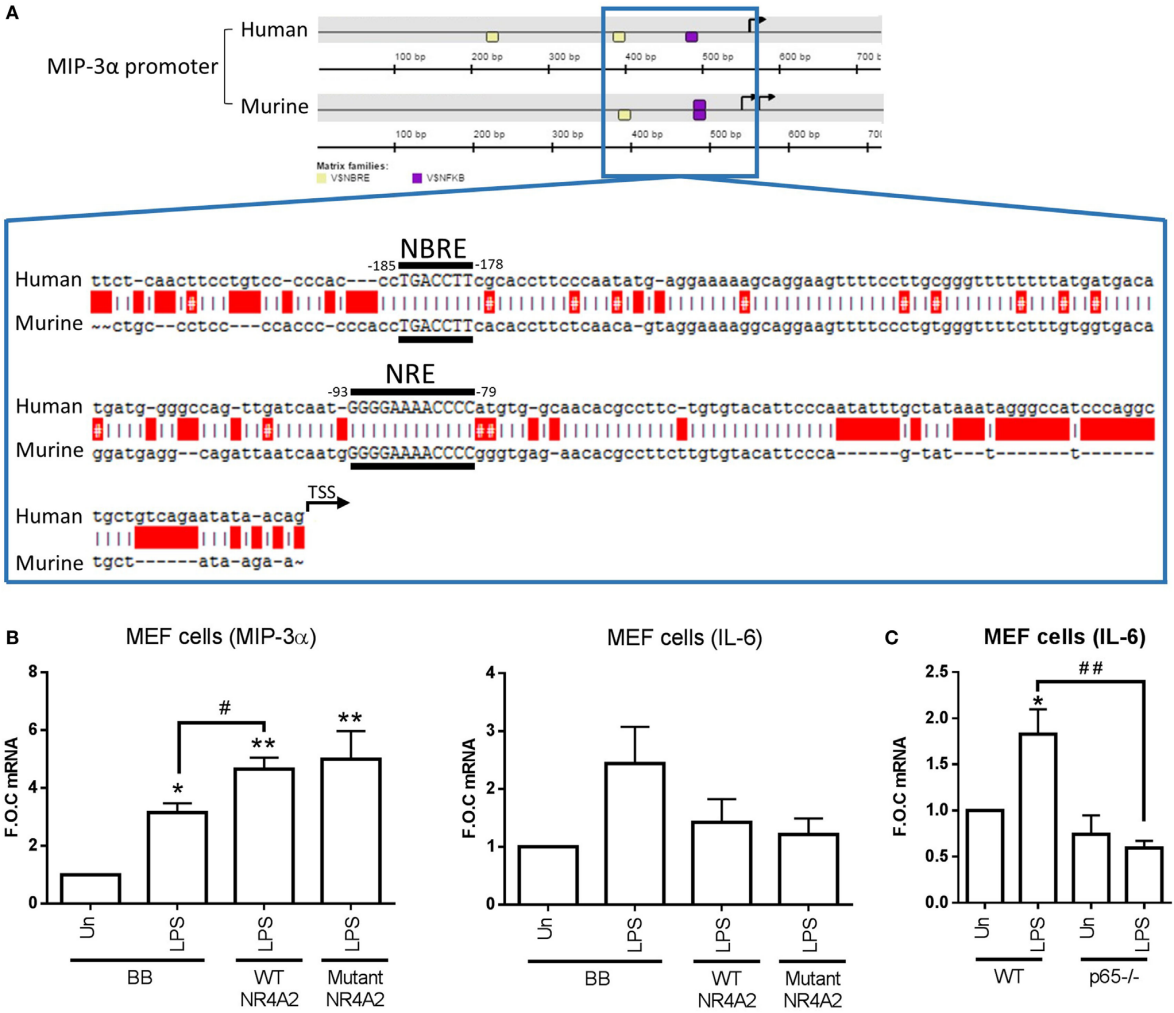
**NR4A2 does not require DNA binding in order to regulate TLR4-driven MIP-3α**. **(A)** Graphical representation [showing ≈500 bp upstream and ≈200 downstream of the transcription start site (TSS) (TSS is indicated on graphic as a black arrowhead)] of the NR4A (shown as yellow box) and NF-κB (shown as purple box) DNA-binding motifs (NBRE and NRE, respectively) on the MIP-3α human and murine promoter analyzed using the Genomatix software suite program MatInspector for 1,000 bp upstream of the TSS. Expanded out regions marked by a blue lined box is ≈200 bp upstream of TSS showing the exact sequence of the NR4A and NF-κB-binding motifs. Human and murine promoter are shown aligned using the ApE plasmid editor software, red boxes indicate gaps, and red boxes with a # indicate a mismatch. **(B)** Mouse embryonic fibroblast (MEF) cells were transfected with 500 ng of wild-type (WT) p-CMX-NR4A2, p-CMX-NR4A2 mutant (C283G), and backbone control plasmid (BB). Following 48 h incubation, cells were treated with 1 µg/ml lipopolysaccharide (LPS) for 2 h. **(C)** MEF cells lacking p65^−/−^ and WT controls were exposed to 1 µg/ml LPS for 2 h. Analysis: RNA was isolated and subsequent qRT-PCR performed to assess levels of MIP-3, IL-6, and control gene β-actin **(B,C)**. Data are expressed as fold over untreated control (FOC) ± SEM for *n* = minimum of 2 **(B)** and 6 **(C)** individual experiments. *p < 0.05, **p < 0.01 treatments compared to untreated control (Un). ^#^*p* < 0.05 treatments compared displayed here using a bar attachment.

In order to investigate how NR4As were positively regulating MIP-3α expression in both human and murine, we performed promoter analysis using the Genomatix MatInspector software suite. Figure 4A shows a graphical representation obtained from Genomatix showing two NBRE (NR4A-binding motif) and one κB (NF-κB-binding motif) site on the human MIP-3α proximal promoter, and on NBRE and κB site on the murine promoter. Intriguingly, no NBRE sites were identified in the promoters of the negatively regulated NR4A target genes IL-12P70 (analyzed IL-12p40 and p35 subunits), IL-10, TNF-α, IL-6, and MCP-1, while one NBRE site was identified in IL-1β (data not shown). Expanded from this graphic is the sequence of both human and murine showing the NBRE and κB sites identified nearest the TSS of both promoters (Figure 4A). We and others have previously shown that p65 is capable of binding to this κB site nearest the TSS (14, 26).

Following this, we investigated whether NR4A receptors needed to bind their NBRE motif in order to enhance MIP-3α expression. Using MEF cells, we overexpressed p-CMX-NR4A2 WT plasmid and a mutant p-CMX-NR4A2 (C283G) plasmid, previously published by our group and others, which lacks the ability to bind to the NBRE sequence on DNA (10, 17). MEF cells displayed successful transfection with significant increases in NR4A2 mRNA in both WT and mutant over backbone control (278 ± 105- and 202 ± 49-fold over control, respectively). We reveal that overexpression of WT NR4A2 can enhance MIP-3α gene expression during LPS stimulation and the DNA-binding mutant NR4A2 does not effect this enhancement (Figure 4B). Furthermore, we show that IL-6 expression due to LPS stimulation is suppressed in both WT and mutant NR4A2 (Figure 4B). Importantly, we confirm using the MEF cells lacking p65^−/−^ that LPS-driven IL-6 in these cells is NF-κB dependent (Figure 4C). Thus, NR4A2 does not require the ability to bind to DNA in order to enhance or repress NF-κB target gene expression.

### NR4A2 and 3 Are Positive Regulators of NF-κB Family Member Relb during TLR4 Stimulation

**Figure 5:**
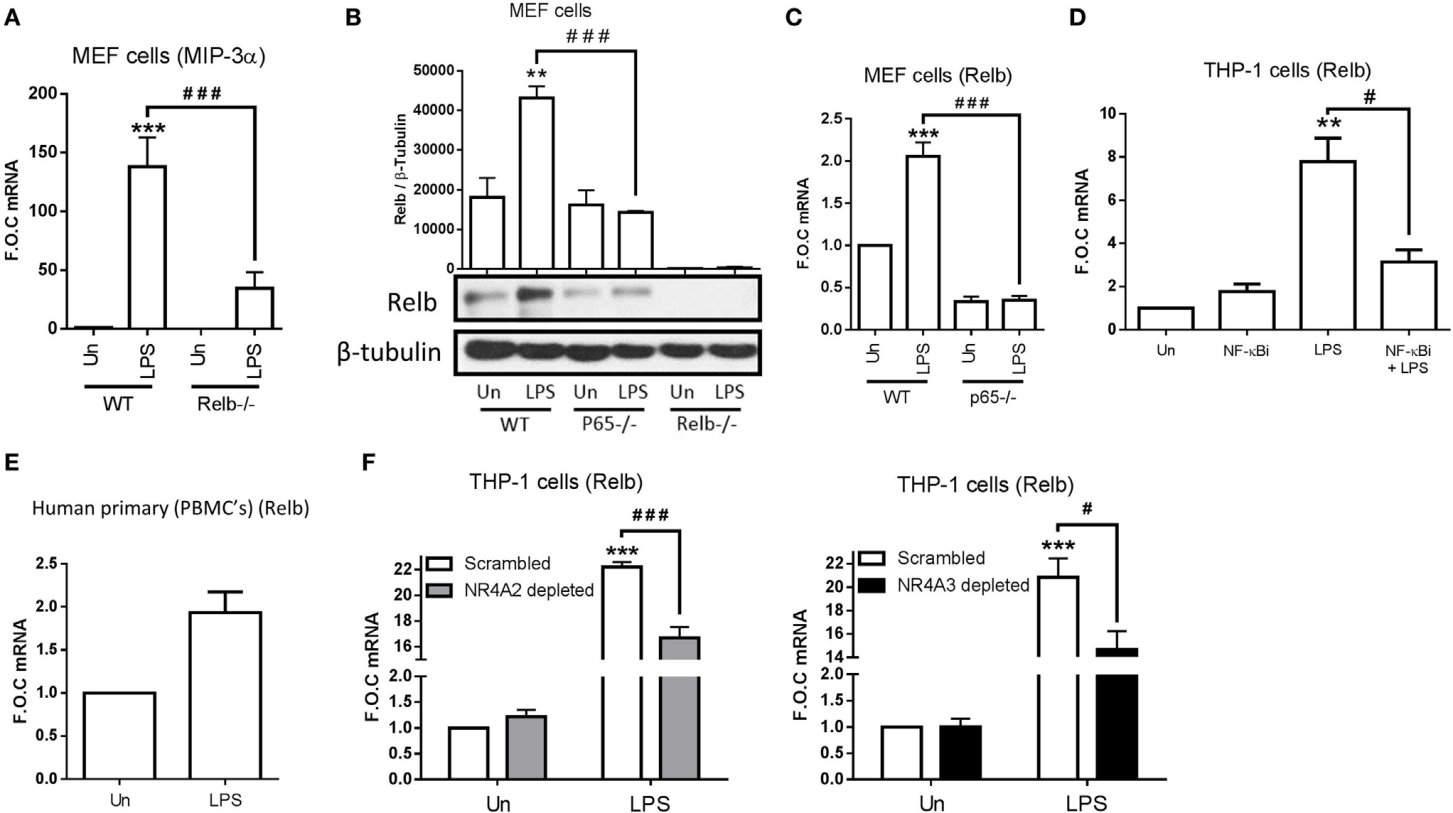
**NR4As are regulators of Relb expression during TLR4 stimulation**. **(A)** Mouse embryonic fibroblast (MEF) cells lacking p65^−/−^ and wild-type (WT) control were exposed to 1 µg/ml lipopolysaccharide (LPS) for 2 h. **(B)** MEF cells lacking p65^−/−^, Relb^−/−^, and WT control were exposed to 1 µg/ml LPS for 4 h. **(C)** MEF cells lacking Relb^−/−^ and WT control were exposed to 1 µg/ml LPS for 2 h. **(D)** Undifferentiated THP-1 cells were pretreated with 10 µM NF-κB inhibitor (NF-κBi) for 1 h followed by the addition of 1 µg/ml LPS for 2 h. **(E)** Human primary PBMCs were exposed to 1 µg/ml LPS for 2 h. **(F)** Undifferentiated THP-1 cells transduced with shRNA directed against scrambled non-target control, NR4A2, or NR4A3 were treated with 1 µg/ml LPS for 2 h. Analysis: RNA was isolated and RT-PCR was performed at indicated times to assess levels of MIP-3α, Relb, and control gene GAPDH **(A,C–F)**. Whole-cell lysates were prepared at indicated time followed by Western blot analysis performed for both Relb and loading control β-tubulin **(B)**. Densitometric analysis included for Western blot data was determined using LI-COR^®^ Image Studio Lite version 3.1, and band density of target protein (Relb) normalized to loading control (β-Tubulin) are displayed above relevant treatments. Un, untreated control. Data are expressed as fold over untreated control (FOC) ± SEM for *n* = minimum of three individual experiments. **p < 0.01, ***p < 0.001 treatments compared to untreated control (Un). ^#^*p* < 0.05, ^###^*p* < 0.001 treatments compared displayed here using a bar attachment.

Following the knowledge that NR4A2 does not require DNA binding to enhance and repress NF-κB target gene expression, we decided to examine more extensively the regulatory elements involved in MIP-3α production. In order to do this, we used MEF cells lacking the NF-κB family members p65^−/−^, p50^−/−^, p52^−/−^, and Relb^−/−^. We have shown that MIP-3α is NF-κB subunit p65 dependent in MEF cells (Figure 3F); additionally, we show that LPS-driven MIP-3α is also NF-κB subunit p50 dependent (data not shown), clearly identifying that it is a canonical NF-κB target gene. Furthermore, we identify that LPS is still capable of driving MIP-3α expression in MEF cells lacking p52^−/−^ (data not shown), however intriguingly we show cells lacking Relb^−/−^ display significantly attenuated MIP-3α expression (Figure 5A). Given that MIP-3α in these cells is confirmed as a p65-dependent gene, we examined whether Relb was also regulated in this manner. Here, we reveal that LPS stimulation significantly drives Relb protein and gene expression in a NF-κB p65-dependent manner (Figures 5B,C). Additionally, we observe that LPS-driven Relb is NF-κB p50-dependent (data not shown). Figure 5B also includes the Relb^−/−^ MEF cells to confirm that the cells lack Relb expression. Furthermore, we show that LPS can drive Relb expression in THP-1 cells and that this is NF-κB dependent, shown using the NF-κB inhibitor BAY 11-7082 (Figure 5D). We also show that Relb gene expression is induced following LPS stimulation using primary PBMC cells (Figure 5E), confirming that this induction is an observation not limited only to immortalized THP-1 and MEF cells.

Taking into account that NR4As are positive regulators of MIP-3α in THP-1 (NR4A2 and 3), Raw mac 264.7, and MEF cells (NR4A2 only tested), we asked whether they were involved in LPS-driven Relb expression. Here, we reveal for the first time that Relb is a novel NR4A target gene, displaying a significant reduction in expression during LPS stimulation in the both NR4A2 and 3 depleted cells (Figure 5F). Thus, we have identified that MIP-3α is both a NR4A and Relb target gene during LPS stimulation, and further reveal that NR4As are important in the LPS regulation of Relb itself. Importantly, we show that NF-κB subunits p65 and p50 (canonical NF-κB) are upstream of all of these factors.

### NR4A2 and 3 Are Positive Regulators of Fibrotic and Atherogenic Factor CTGF

**Figure 6:**
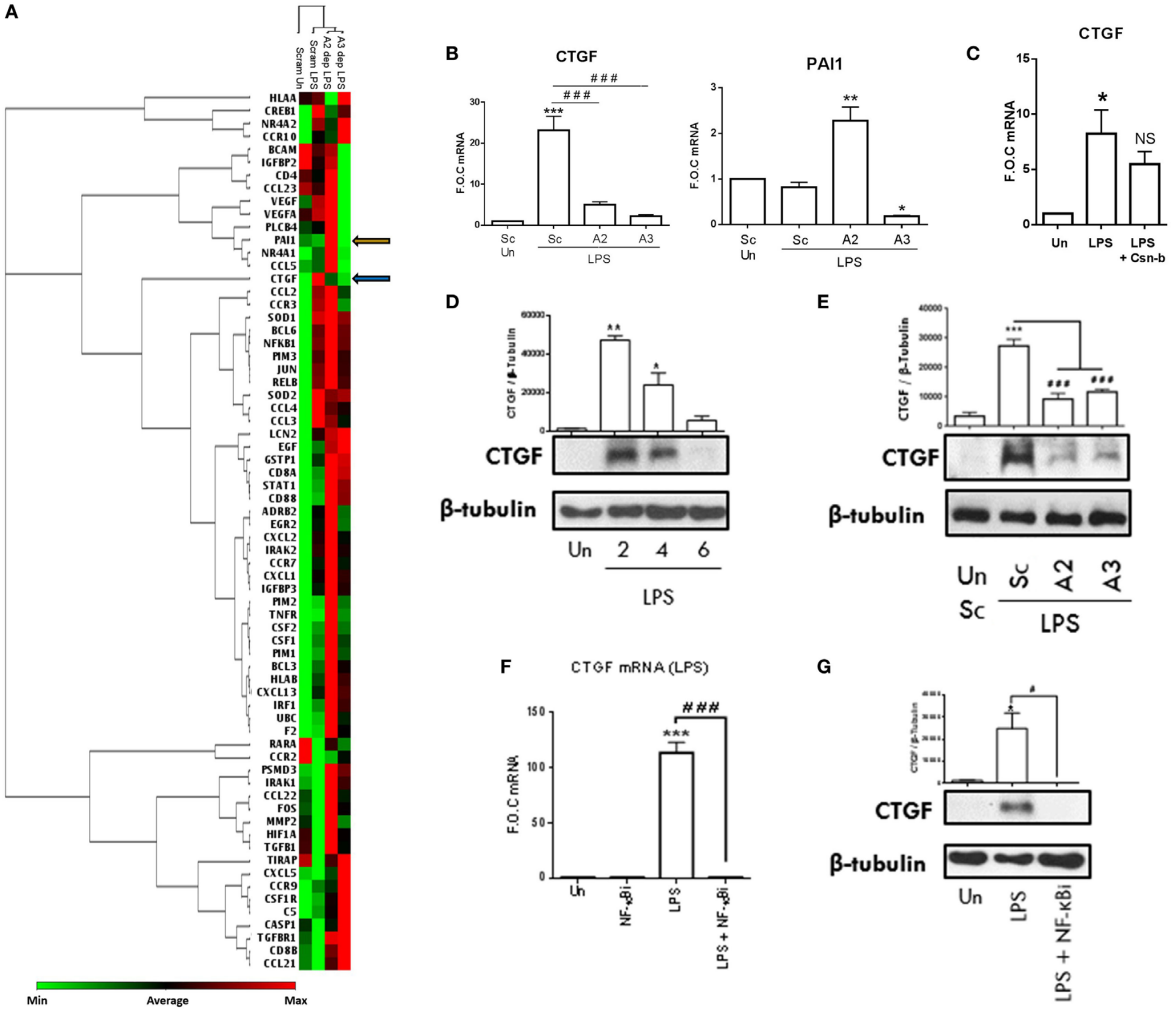
**NR4As are key regulators of inflammatory driven CTGF**. **(A)** Undifferentiated THP-1 cells transduced with shRNA directed against scrambled non-target control (Sc), NR4A2 (A2 dep), or NR4A3 (A3 dep) were treated with 100 ng/ml lipopolysaccharide (LPS) for 2 h. RNA was isolated was from 3 technical replicates, pooled, and a PCR array (consisting of 92 targets and 4 reference genes) was performed. Using a web-based PCR array data analysis tool [SABiosciences, Qiagen], a clustergram was made. A clustergram performs non-supervised hierarchical clustering of the entire data set and is represented as a heat map with a dendrogram showing individual genes and overall treatment groups with a similar regulation pattern. **(B)** Genes of interest were then investigated using qRT-PCR on three separate experiments (*n* = 3) following the same protocol used in the array described above. **(C)** Undifferentiated THP-1 cells were pretreated with 100 nM Cytosporone-B (Csn-B) for 30 min followed by the addition of 100 ng/ml LPS for a further 1.5 h. **(D)** Undifferentiated THP-1 cells were exposed to 1 µg/ml LPS for 0, 2, 4, and 6 h. **(E)** Undifferentiated THP-1 cells transduced with shRNA directed against scrambled non-target control, NR4A2, or NR4A3 were treated with 1 µg/ml LPS for 4 h. **(F,G)** Undifferentiated THP-1 cells were pretreated with 10 µM NF-κB inhibitor (NF-κBi) for 1 h followed by the addition of 1 µg/ml LPS for 2 h **(F)** or 4 h **(G)**. Analysis: **(B,C,F)** RNA was isolated at times indicated and RT-PCR was performed at indicated times to assess levels of CTGF, PAI-1, and control gene GAPDH. Whole-cell lysates were then prepared from all treatments and Western blot analysis was performed for CTGF and loading control β-Tubulin **(D,E,G)**. Data are expressed as fold over untreated control (FOC) ± SEM for *n* = minimum of three individual experiments or representative Western blot for *n* = minimum of three individual experiments. *p < 0.05, **p < 0.01, ***p < 0.001 treatments compared to untreated control (Un). ^###^*p* < 0.001 treatments compared displayed here using a bar attachment. Blue arrow demonstrates where CTGF is on the heat map, and orange arrow indicates where PAI-1 is. Un, untreated control; Dep, depleted.

In order to expand our knowledge of NR4A2/3 regulated target genes during inflammatory pathogenesis, we performed an in-house PCR array. Scrambled control THP-1 cells, NR4A2 and 3 depleted cells were stimulated with LPS (100 ng/ml) for 2 h. A heat map represents changes in gene expression from the array and similarities in the pattern of gene regulation and overall treatment similarities are represented by the inclusion of a dendrogram (Figure 6A). All data obtained from the PCR array analysis are shown in Table S3 in Supplementary Material. Genes of interest were further investigated within the same experimental model and analyzed using qRT-PCR (Figure 6B). LPS significantly drives CTGF in scrambled control THP-1 cells, and this induction is significantly attenuated in NR4A2 and 3 depleted cells (Figure 6B). Intriguingly while LPS treatment alone does not alter PAI-1 expression, an established NR4A1 regulated target gene in human umbilical vein endothelial cells, MCF-7, and JURKAT cells during TNFα stimulation (27), NR4A2 depletion significantly potentiates LPS effects on PAI-1gene expression and NR4A3 depleted cells display significantly reduced PAI-1 levels. Using the NR4A1 agonist Csn-B (100 nM), we reveal that enhanced NR4A1 activity reduces CTGF expression (Figure 6C).

Here, we establish that TLR4 stimulation drives CTGF expression, an important atherogenic and fibrotic factor, and for the first time show NR4A receptor activity is crucial to this induction. To further characterize the mechanisms involved in this regulation, we establish that LPS (1 µg/ml) significantly drives CTGF protein expression as early as 2 h and this induction declines over 4 and 6 h (Figure 6D). Further analysis supports the changes in gene expression using the PCR array, whereby LPS (1 µg/ml) induces CTGF protein expression at 4 h, which is significantly attenuated in NR4A2 and 3 depleted cells (Figure 6E). Using the NF-κB inhibitor BAY 11-7082 (10 µM), we confirm that LPS (1 µg/ml) and TNFα-induced CTGF gene and protein expression is NF-κB dependent (Figures 6F,G; Figure S4 in Supplementary Material), consistent with other LPS-induced targets controlled by NR4A activity including MIP-3α and MCP-1. We next examined whether CTGF was a Relb target gene, similar to that of MIP-3α, the other positively regulated NR4A gene identified in this study. Unfortunately however, examination of the MEF cells reveals no enhancement of CTGF during LPS stimulation, a mechanism that may be cell type specific and therefore we could not investigate the Relb involvement using these MEF Relb^−/−^ cells. Moreover, promoter analysis shows no NR4A DNA-binding motifs (NBRE sites) on the promoter of CTGF (data not shown). Thus, we have identified NR4A receptors regulate novel target gene CTGF, an important fibrotic and atherogenic target gene.

## Discussion

**Figure 7:**
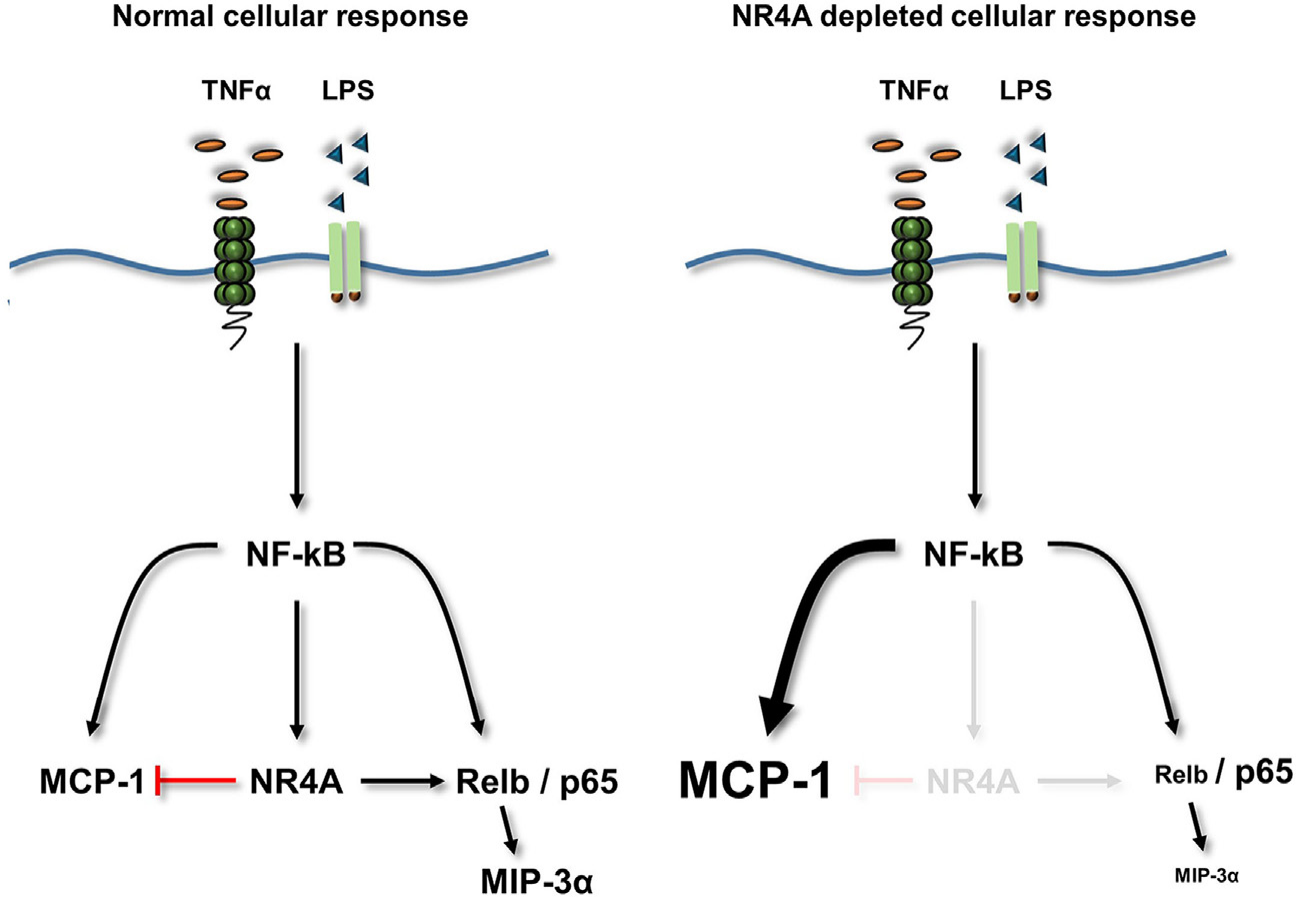
**Summary diagram**. TLR4 or TNFα receptor stimulation increases the activity of NF-κB and the expression of its target genes NR4A1–3 (NR4A), MCP-1, MIP-3α, and Relb. Loss of NR4A potentiates this induction of MCP-1, while reducing the induction of Relb and MIP-3α. Furthermore using mouse embryonic cells, we identify MIP-3α is a novel Relb target gene. Therefore, NR4A receptors act as repressors of inflammatory-NF-κB-driven MCP-1 (indicated by a red T bar) and enhancers of NF-κB-driven Relb and subsequently MIP-3α. Using a NR4A2 DNA-binding mutant reveals that NR4A2 does not require the ability to bind DNA in order to enhance/repress target genes simultaneously.

Dysregulation of an appropriate inflammatory response plays a critical role in the pathogenesis of diseases such as atherosclerosis and rheumatoid arthritis. Using NR4A depleted human and murine myeloid cells, alongside MEF cells overexpressing NR4A2, we reveal that these receptors are capable of dynamic regulation of NF-κB target genes including MIP-3α and MCP-1, as summarized in Figure 7. NR4A receptors negatively regulate MCP-1 and positively regulate key inflammatory chemokine MIP-3α simultaneously during TLR4 or TNFR stimulation. Employing an inflammatory PCR array, we reveal a new target gene CTGF, a key fibrotic and atherogenic factor, to be positively regulated by the NR4A receptors. Intriguingly, both NR4A3 and MIP-3α have been shown to play crucial roles in the pathogenesis of atherosclerosis, and here, we show an enhancement and correlation in gene expression of both in carotid plaques from symptomatic patients. Mechanistic investigations reveal that NR4A2 does not require DNA binding in order to simultaneously enhance or repress NF-κB target gene expression. Furthermore, we reveal the NF-κB family member Relb to be a novel NR4A target gene during TLR4 stimulation and that Relb is critical in the regulation of MIP-3α. Thus, we reveal a complex dynamic, whereby NR4A receptors can simultaneously enhance or repress NF-κB target gene expression, and that they do not need to bind DNA in order to do so and Relb, acting as a novel NR4A target, may be important in this dynamic system.

Within the nuclear hormone receptor family, the NR4A receptor subfamily, which includes NR4A1, NR4A2, and NR4A3, has emerged as key regulators of inflammatory responses (1–3). NR4A family members attenuate LPS and TNFα stimulated pro-inflammatory response in myeloid cells principally limiting NF-κB activation (3). We and others have shown that NR4A expression is increased by LPS stimulation in myeloid cells (14, 18). Here, we support these observations showing that LPS stimulation drives all three NR4A genes in primary and immortalized human and murine monocyte/macrophage cells. Within this study, we further reveal that LPS-driven NR4A genes in human and murine myeloid cells is NF-κB dependent. We have recently reported that depletion of NR4A2 and 3 in human monocyte cells results in a hyper-pro-inflammatory cell phenotype, displaying enhanced NF-κB target genes TNFα and MCP-1 protein secretion during LPS stimulation (14). This study supports and expands these observations showing that NR4A2 and 3 depletion in myeloid cells results in enhanced NF-κB-driven IL-12p70, Il-1β, IL-10, TNF-α, IL-6, and MCP-1 gene expression. The repressive mechanism of NR4As has been examined revealing NR4As recruit co-repressor complex CoREST to NF-κB on its target genes, subsequently limiting its transcriptional activity (11). Collectively, we describe how NR4A genes are regulated by NF-κB in order to feedback/limit downstream target gene expression. This study therefore expands and supports growing observations in several cell types deciphering this feedback regulatory loop, which is needed to control the magnitude of inflammatory responses (11, 12, 14, 19).

Intriguingly, NR4A receptors can enhance NF-κB activity and subsequent target gene expression, with NR4A2 enhancing IL-8 through cooperation with p65 in rheumatoid arthritic synoviocyte cells (10). Maijenburg et al. also revealed through gain of function experiments that NR4A1 and 2 can increase both IL-8 and IL-6 production in mesenchymal stromal cells, albeit the role of NF-κB was not determined (28). Within this study, we demonstrate that LPS and TNFα stimulation drives gene and protein secretion of chemokines MIP-3α and MCP-1 in primary and immortalized human and murine (LPS only) myeloid cells. We further identify NF-κB to be the upstream regulator of both chemokines within these conditions and cells consistent with previous studies in myeloid and other cell types (14, 26, 29, 30). Interestingly, we observed using depleted cells that NR4A2 and 3 were positive regulators of inflammatory-driven MIP-3α in human THP-1 cells and murine RAW264.7 macrophage cells (NR4A2 only), while repressors of MCP-1 as stated above. Additionally, overexpression of NR4A2 in MEF cells supports its role as a regulator of LPS driven MIP-3α expression.

We have recently shown that adenosine drives NR4A receptor expression in myeloid cells, primarily through the A2a receptor (14). Furthermore, THP-1 cells exposed to LPS or TNFα and subsequently treated with adenosine receptor A2a agonist display increased NF-κB-dependent MIP-3α expression. Analysis revealed that depletion of NR4A2 within this model results in further enhancement of MIP-3α expression with concurrent NF-κB/p65 binding to its promoter (14). Additionally, using vascular SMCs, it has recently been shown that overexpression of NR4A3 can attenuate LPS-driven MIP-3α production (19). Importantly, herein we reveal that NR4A2 and 3 can act as positive regulators of MIP-3α expression in human and murine myeloid cells during TLR4 or TNFα receptor stimulation, and furthermore, this regulation is governed by NF-κB activity. Promoter analysis reveals NR4A and NF-κB-binding motifs (NBRE and NRE, respectively) on the MIP-3α promoter in both human and murine species. Taking this into account, we investigated whether NR4A receptors are needed to bind to the MIP-3α promoter, at their consensus motif in order to positively regulate its expression. Using a mutant NR4A2 plasmid (C293G), which lacks the ability to bind DNA (10, 17), alongside a WT control, we revealed that NR4A2 did not require the ability to bind DNA in order to enhance LPS driven MIP-3α, while simultaneously repressing NF-κB target gene IL-6. The ability of NR4A receptors to enhance gene expression while not binding DNA has been shown previously and we support these observations (10).

In order to gain more mechanistic insight, we utilized MEF cells lacking all NF-κB family members p65, p50, p52, and Relb. We reveal that LPS-induced MIP-3α is not only p65 regulated but also p50 (displaying canonical pathway regulation) and more interestingly Relb dependent. Moreover, we show that Relb is induced by LPS in MEF and myeloid cells and that this regulation is dependent upon canonical NF-κB family members. Herein, we support previous studies showing inflammatory mediators such as LPS or TNFα can induce Relb in myeloid cells, and some studies revealing the canonical NF-κB family are involved in this regulation (31–34). We reveal that NR4A depletion in THP-1 cells results in attenuated MIP-3α with enhanced IL-6 protein secretion, and overexpression of NR4A2 in MEF cells shows the inverse during LPS stimulation. Intriguingly, Tully et al. revealed in lung epithelial cells that Relb depletion resulted in enhanced IL-6 production alongside attenuated MIP-3α production (32). Here, we show this phenomenon is paralleled in NR4A-depleted cells, and furthermore, we reveal Relb to be a novel NR4A target gene during LPS stimulation. Thus, this study highlights a more complex model of NR4A–NF-κB interaction in myeloid cells. Highlighting the ability for NR4A2 and 3 to enhance and repress NF-κB target genes simultaneously, independent of DNA binding, in same cell types exposed to distinct pro-inflammatory stimuli. Finally, we show Relb to be a novel NR4A target gene during LPS responses, and given that it can also enhance/repress inflammatory genes simultaneously, we speculate this may be a component of how NR4As are performing such an action.

In order to expand our analysis, with the aim of identifying more NR4A positively/negatively regulated genes during LPS stimulation, we utilized a PCR array consisting of 92 inflammatory genes. From this evaluation, we observed NR4A2 and 3 to be positive regulators of key fibrotic and atherogenic factor, CTGF (35–38) during LPS stimulation. Using bronchial epithelial cells, LPS has previously been shown to regulate CTGF through NF-κB (39). Within this study, we reveal for the first time, to our knowledge, LPS stimulated CTGF in myeloid cells is NR4A2 and 3 dependent and is regulated as early as 2 h and declines by 6 h, prototypical of an immediate early gene response. Further analysis reveals that this regulation is co-ordinated by NF-κB, which supports previous studies using bronchial epithelial cells (39). Recent evidence has emerged highlighting the pivotal role NR4A1 plays in fibrosis, specifically impacting on the TGFβ1 pathway (40), given CTGF is an important factor influencing both fibrosis and the TGFβ1 pathway (35, 36), we speculate NR4A2 and 3 may also play roles in fibrotic disease and is currently under investigation in our laboratory.

Thus, NR4A receptors are key regulators of inflammatory signaling, primarily modulating NF-κB activity. Here, we reveal NR4As are capable of simultaneously directing NF-κB target gene expression, enhancing MIP-3α and CTGF while repressing MCP-1 and other major inflammatory chemokines and cytokines. We reveal novel data showing NR4As can regulate LPS-driven Relb and that Relb is involved in LPS-driven MIP-3α. Additionally, we show that NR4As do not require the ability to bind DNA in order to simultaneously enhance and repress NF-κB target gene expression. Such an observation identifies a greater complexity to how NR4A receptors can effect NF-κB signaling. This study expands on and highlights the critical role NR4A nuclear receptor play in regulating key inflammatory genes regulated by the NF-κB family.

## Author Contributions

CM performed experiments and contributed to study design and manuscript preparation. MG performed experiments and manuscript preparation. HG performed experiments and manuscript preparation. BB performed experiments and manuscript preparation. EC performed experiments and manuscript preparation. EB performed experiments and manuscript preparation. MB performed carotid endarterectomy on patients. OB contributed to study design and manuscript preparation. CG contributed to study design and manuscript preparation. EM contributed to study design and manuscript preparation. DC conceived and designed study and prepared manuscript.

## Conflict of Interest Statement

The authors declare that the research was conducted in the absence of any commercial or financial relationships that could be construed as a potential conflict of interest.
