# Supplementary material for: NR4A Receptors Differentially Regulate NF-κB Signaling in Myeloid Cells
Source: Front Immunol. 2017 Jan 23;8:7. doi: 10.3389/fimmu.2017.00007 (PMC5256039; doi:10.3389/fimmu.2017.00007)
Supplement: Supplementary file 1 [file Table_1.PDF]

Patient ID			Morphological parameters			Symptomatic status			Carotid stenosis			Hypercholesterolemia (HPC)					
												Fasting lipid profile					Chol. Lowering therapy
Sample #	Age	Sex	Weight (kg)	Height (cm)	BMI	Status	Past CAD events	Time of event	Left (%)	Right (%)	CEA side	Tot Chol (mmol/l)	LDL (mmol/l)	HDL (mmol/l)	Triglycerides	Diagnosis	Statins/dose (OD)
1	64	M	NA	NA	NA	**Asymptomatic**	None	NAPP	60	Normal	Left	NA	NA	NA	NA	HPC	Rosuvastatin / 10mg
2	71	M	85.6	172	29.3	**Asymptomatic**	None	NAPP	> 75	> 70	Left	5	1.14	3.57	0.64	HPC	Rosuvastatin / 10mg
3	58	M	85	183	25	**Asymptomatic**	None	NAPP	> 99	Normal	Left	4.7	2.2	1.2	2.9	HPC	Atorvastatin / 20mg
4	78	F	58.3	151	25	**Symptomatic**	Stroke	6 weeks before CEA	Normal	80-90	Right	5.4	2.52	2.71	0.38	HPC	Atorvastatin / 20mg
5	63	M	71.3	NA	NA	**Symptomatic**	TIA + MI	same year as CEA	NA	> 95	Right	4.4	2.44	1.67	0.06	HPC	Atorvastatin (lipitor) / 40mg OD
6	75	M	75.6	NA	NA	**Symptomatic**	Stroke	NA	NA	99	Right	5.7	NA	NA	NA	HPC	Atorvastatin (lipitor) / 80mg OD

Patient ID	Diabetes			Hypertension (HTN)		
		time from	Anti-Diabetic therapy		Antihypertensive therapy	Anti-platelets therapy
Sample #	Diagnosis	diagnosis	antidiabetic drug/dose (OD)	Diagnosis	Antihypertensive drug/dose (OD)	ASA/dose
1	type 2	6 months	Metformin/500mg	HTN	Amlodipine / 10mg + Bisopralol / 5mg	Aspirin / 75mg OD
2	No	NAPP	No	HTN	Amlodipine / 10mg + Furosemide / 20mg + Oimesartan 10mg + Doxasin / 4mg	No
3	type 2	3 years	Insulin / 8units	HTN	Perindopril / 10mg + Losartan / 12.5mg	Aspirin / 75mg OD
4	No	NAPP	No	HTN	Perindopril/Indapamide / 2.5mg	Aspirin / 75mg OD
5	No	NAPP	No	HTN	Perindopril / 7.5mg + Bendroflumethiazide / 1.25mg	Aspirin / 75mg OD
6	No	NAPP	No	HTN	Valsartan / 40mg + Bendroflumethiazide / 2.5mg	Aspirin / 75mg OD

Patient ID	Lifestyle								
	Smoking habits					Alcohol intake		Physical activity	
Sample #	Smoking status	cigarettes/day	pack/year	duration of smoking	durations of cessation	Std unit/week	abuse	Sedentarity	min/day
1	NA	NA	NA	NA	NA	NA	NA	NA	< 30
2	NA	NA	NA	NA	NA	NA	NA	NA	NA
3	Current	20	46	--	--	18	In the past	NA	NA
4	Current	5	NA	--	--	NA	NA	No	> 30
5	ex	15-20	NA	NA	5 weeks	40	NA	NA	NA
6	Current	--	--	--	--	NA	NA	NA	NA

Supplemental Table 1: Relevant clinical patient data. NA = Not available, NAPP = Not applicable, TIA = Transient Ischemic attack, MI = myocardial infarction, CAD = Coronary artery disease, CEA = Carotid endarterectomy
