# Supplementary material for: NR4A Receptors Differentially Regulate NF-κB Signaling in Myeloid Cells
Source: Front Immunol. 2017 Jan 23;8:7. doi: 10.3389/fimmu.2017.00007 (PMC5256039; doi:10.3389/fimmu.2017.00007)
Supplement: Supplementary file 2 [file Table_2.PDF]

Human	Forward primer	Reverse primer
GAPDH	CGACAGTCAGCCGCATCTT	CCCCATGGTGTCTGAGCG
NR4A1	GTTCTCTGGAGGTCATCCGCAAG	GCAGGGACCTTGAGAAGGCCA
NR4A2	TATTCCAGGTTCCAGGCGAA	GCTAATCGAAGGACAAACAG
NR4A3	CCAAGCCTTAGCCTGCCTGTC	AGCCTGTCCCTTACTCTGGTGG
MIP-3α	CTGGCTGCTTTGATGTCAGT	CGTGTGAAGCCCACAATAAA
MCP-1	CTGCTCATAGCAGCCACCTT	CAGGTGACTGGGGCATTG
PAI-1	AGTGGACTTTTCAGAGGTGGA	GCCGTTGAAGTAGAGGGCATT
Relb	Taqman primer set used	Taqman primer set used
CTGF	Taqman primer set used	Taqman primer set used
**Murine**	**Forward primer**	**Reverse primer**
GAPDH	TGTGTCCGTCGTGGATCTGA	CCTGCTTCACCACCTTCTTGA
NR4A1	ATGCCTCCCCTACCAATCTTC	CACCAGTTCCTGGAACTTGGA
NR4A2	TCAGAGCCCACGTCGATT	TAGTCAGGGTTTGCCTGGAA
NR4A3	TTAACCCATGTCGCTCTGTG	TGCAGAGCCTGAACCTTGAT
MIP-3α	TCTGCTCTTCCTTGCTTTGGC	AGTCGTAGTTGCTTGCTTCTGC
MCP-1	GCCTGCTGTTCACAGTTGC	GAGTGGGGCGTTAACTGCAT
IL-6	GTCACAGAAGGAGTGGCTA	AGAGAACAACATAAGTCAGATACC
Relb	Taqman primer set used	Taqman primer set used
β-actin	CTAGGCACCAGGGTGTGAT	TGCCAGATCTTCTCCATGTC

Supplemental Table 2

RTPCR primer sequences
