# Supplementary material for: NR4A Receptors Differentially Regulate NF-κB Signaling in Myeloid Cells
Source: Front Immunol. 2017 Jan 23;8:7. doi: 10.3389/fimmu.2017.00007 (PMC5256039; doi:10.3389/fimmu.2017.00007)
Supplement: Supplementary file 3 [file Table_3.pdf]

		Raw CT value				Fold over control		
		Sc Un	Sc LPS	A2 LPS	A3 LPS	Sc LPS	A2 LPS	A3 LPS
1	CXCL2	31.14992	22.73847	22.36268	23.26534	270.1479	477.8786	230.6385
2	CCL4	29.2163	21.26891	22.25187	22.41418	195.8424	135.0813	108.9129
3	CCL3	30.00071	22.1717	22.97505	23.57886	180.4138	140.9384	83.6769
4	SOD2	25.53984	18.91711	19.76765	19.50155	78.1887	59.1148	64.1423
5	CXCL1	29.03405	23.61576	23.15389	23.74967	33.9289	63.7086	38.0358
6	JUN	26.79071	22.32501	22.33466	22.86494	17.5312	23.7409	14.8323
7	CTGF	26.72243	22.46014	24.33148	25.69795	15.2259	5.6734	1.9853
8	CCL2	31.33692	27.42727	27.5765	28.71222	11.9241	14.6586	6.0193
9	NR4A2	28.32638	24.77977	26.08987	24.76966	9.2713	5.0974	11.4846
10	ADRB2	27.35419	24.51102	24.06017	25.40049	5.6935	10.6094	3.7806
11	BCL6	27.39085	24.65551	24.67729	24.94376	5.2835	7.0951	5.3221
12	EGR2	29.07135	26.42558	25.93274	27.30603	4.9655	9.526	3.3178
13	RELB	24.75997	22.19641	22.30392	22.66935	4.6904	5.9352	4.157
14	IRAK2	27.98981	25.44953	25.2757	25.8333	4.6153	7.0978	4.3512
15	IGFBP3	28.20742	25.77161	25.26742	25.81345	4.2929	8.3008	5.1297
16	NFKB1	24.60679	22.17247	22.27395	22.53165	4.2885	5.4494	4.1126
17	BCL3	26.49616	24.54499	23.58496	24.20171	3.0681	8.1367	4.7878
18	PIM3	23.39751	21.53365	21.5566	21.91324	2.8879	3.8749	2.7305
19	CCR7	29.53502	27.69137	27.28666	27.96899	2.8477	5.1395	2.8897
20	CSF1	29.4992	27.68489	26.5548	27.56183	2.7904	8.3262	3.738
21	CREB1	26.24313	24.44304	25.7768	25.08096	2.763	1.4944	2.1842
22	SOD1	26.60117	24.92122	25.2939	25.36507	2.5422	2.6768	2.299
23	HLAB	24.15229	22.80569	22.32429	22.65917	2.0178	3.8404	2.7473
24	CSF2	33.74995	32.74287	31.83027	32.8361	1.5946	4.0924	1.8388
25	PIM1	27.0869	26.12467	25.16558	26.02549	1.5458	4.097	2.0368
26	IRF1	24.44476	23.53282	22.82708	23.12451	1.4929	3.3194	2.4371
27	LCN2	29.83711	28.93657	29.15518	28.93227	1.4811	1.7353	1.8273
28	CXCL13	28.55065	27.75372	27.75275	27.86095	1.3785	1.8806	1.5742
29	CCR3	28.71318	27.91974	28.29062	28.55029	1.3752	1.4498	1.0926
30	GSTP1	21.85443	21.06199	21.01799	20.93047	1.3742	1.9315	1.8517
31	NR4A1	27.45214	26.68767	26.47102	27.36009	1.3478	2.1352	1.0403
32	VEGF	25.0445	24.29543	24.65013	25.26309	1.3335	1.4217	0.8388
33	STAT1	24.4347	23.73359	23.10971	23.18428	1.2899	2.7099	2.3219
34	EGF	29.49569	28.82897	28.73617	28.48778	1.2595	1.8312	1.9627
35	CCR10	28.78988	28.15419	28.68277	28.21911	1.2327	1.165	1.4496
36	PLCB4	33.26122	32.64249	32.23985	35.27039	1.2183	2.1956	0.2924
37	CCL5	23.90444	23.29534	22.85884	24.26637	1.2102	2.2328	0.7594
38	CCR9	28.76031	28.17448	28.48203	27.92778	1.1908	1.3118	1.738
39	CSF1R	25.85252	25.28747	25.26379	24.69856	1.1738	1.6267	2.1718
40	HLAA	23.37243	22.82101	25.91243	22.65352	1.1628	0.186	1.6064
41	VEGFA	24.6753	24.15375	24.52227	25.18519	1.139	1.2027	0.6854
42	PIM2	24.49816	23.98961	23.266	23.95461	1.1287	2.541	1.4225
43	CD88	28.77457	28.27883	27.92855	27.95474	1.1188	1.9443	1.7228
44	CD8A	34.2394	33.76821	33.78652	33.69176	1.0999	1.4806	1.4266
45	C5	26.77764	26.31187	26.54886	26.14644	1.0958	1.2675	1.5116

46	UBC	19.86549	19.40108	19.10241	19.3764	1.0947	1.8357	1.3698
47	F2	29.19934	28.80787	28.67804	28.81088	1.0408	1.5525	1.2775
48	TNFR	27.07616	26.72774	26.77651	26.94088	1.0102	1.3314	1.0719
49	CD4	23.19196	22.91118	23.21299	23.36378	0.9639	1.066	0.8664
50	CXCL5	29.27982	29.01321	29.22351	28.74011	0.9545	1.1247	1.4188
51	PSMD3	22.96623	22.75305	22.31168	22.36795	0.9198	1.7027	1.4775
52	IRAK1	22.08351	21.89395	21.6256	21.71109	0.9048	1.4857	1.2634
53	CD8B	27.92932	27.74818	27.79096	27.51084	0.8996	1.1905	1.3044
54	CCL23	25.95272	25.79071	25.93948	26.72124	0.8877	1.0916	0.5729
55	TGFBR1	23.2707	23.20074	22.95317	22.78349	0.8328	1.348	1.368
56	CCL21	27.85966	27.80712	27.61073	27.1968	0.8228	1.2854	1.5452
57	BCAM	28.91295	28.86309	29.15332	29.90005	0.8213	0.9157	0.4924
58	CCL22	27.0687	27.08534	26.71331	26.95792	0.7843	1.3838	1.0539
59	IGFBP2	23.35605	23.40438	23.55452	24.39974	0.7673	0.9426	0.4734
60	HIF1A	23.80524	23.85501	23.72005	23.82723	0.7665	1.1475	0.9612
61	MMP2	26.22093	26.41629	25.82162	26.38552	0.6929	1.4266	0.8707
62	PAI1	26.95213	27.16122	25.2548	29.09395	0.6864	3.5079	0.2211
63	TGFB1	26.16486	26.37853	26.03129	26.23649	0.6842	1.1866	0.9287
64	CASP1	26.03423	26.29567	26.09057	25.42818	0.6619	1.0402	1.4855
65	TIRAP	29.55846	30.03474	29.84398	29.42449	0.5703	0.8875	1.0709
66	FOS	24.79805	25.28494	23.99652	24.45	0.5662	1.8853	1.2423
67	RARA	24.13761	24.94797	24.60934	24.86743	0.4524	0.78	0.5885
68	CCR2	24.55592	25.99046	26.03858	25.18642	0.2935	0.3871	0.6304
69	TBP	24.90204	24.59749	24.54666	24.69837			
70	HPRT1	24.72117	23.98555	25.93588	24.83725			
71	GAPDH	18.69646	18.5585	18.43142	18.80203			
72	ACTB	16.37471	16.21746	16.23345	16.21637			
73	IL5	35.05675	35.40499	Undeterm	Undeterm	0.7934	1.0817	0.976
74	MUC5AC	35.07878	33.48107	34.21128	34.25095	2.2738	1.8686	1.6403
75	IL15	35.08383	34.34113	32.40438	35.99188	1.2527	6.5381	0.976
76	RETN	35.25301	33.32288	33.91895	34.45923	2.5373	2.2883	1.4198
77	CCL11	35.62135	33.73069	37.50751	Undeterm	1.9125	1.0817	0.976
78	CCL18	35.65987	32.85978	32.41666	33.97801	3.4976	6.4827	1.9819
79	CCL8	36.23048	32.90314	31.7187	33.23433	3.3941	10.5163	3.3186
80	CXCL3	36.5421	25.40423	24.795	25.98067	613.9275	1276.75	506.4362
81	MMP3	36.81456	38.50647	36.78519	37.38254	0.7934	1.0817	0.976
82	CCL19	36.81662	33.65831	34.43886	32.83365	2.0109	1.5959	4.381
83	PLAT	36.91149	34.43683	31.85399	33.53905	1.1723	9.575	2.6867
84	CCL7	37.02137	35.30249	34.82135	34.53918	0.7934	1.2243	1.3433
85	CXCL3	37.36361	25.19321	24.83692	25.97578	710.6249	1240.179	508.1567
86	IL24	37.89324	34.74892	34.88383	37.90767	0.9442	1.1724	0.976
87	EDN1	39.52799	32.47358	32.37841	33.58132	4.5712	6.6569	2.6092
88	IGFBP1	39.53119	Undeterm	Undeterm	Undeterm	0.7934	1.0817	0.976
89	CCL13	39.73958	37.61808	35.56335	38.72113	0.7934	1.0817	0.976
90	TGFB2	39.85622	Undeterm	Undeterm	Undeterm	0.7934	1.0817	0.976
91	NOD2	Undeterm	Undeterm	39.51672	Undeterm	0.7934	1.0817	0.976
92	VCAM1	Undeterm	34.01697	30.35393	31.14284	1.5683	27.0832	14.1436
93	RELMB	Undeterm	37.94013	39.71878	39.73948	0.7934	1.0817	0.976
94	PCK1	Undeterm	36.00609	Undeterm	Undeterm	0.7934	1.0817	0.976
95	BCMA	Undeterm	Undeterm	Undeterm	Undeterm	0.7934	1.0817	0.976
96	EGFR	Undeterm	32.45424	33.60786	36.97974	4.6329	2.839	0.976

Supplemental Table 3: PCR array gene expression analysis. Undifferentiated THP-1 cells transduced with shRNA directed against scrambled non target control (Sc), NR4A2 (A2) or NR4A3 (A3) were treated with LPS (100ng/ml) for 2hrs. RNA was isolated from 3 technical replicates, pooled and a PCR array (consisting of 92 target genes and 4 reference genes) was performed for each treatment. Displayed here are raw CT values, fold over untreated (Un) control values (calculated as described in materials and methods). Values in red are those that changed ≥ 2 fold greater than Sc Un. Values in blue are those that changed ≥ 2 fold less than Sc Un. Reference genes are highlighted yellow and genes whose Sc Un had CT values ≥ 35 and deemed unreliable are highlighted in green.
