# Supplementary material for: NR4A Receptors Differentially Regulate NF-κB Signaling in Myeloid Cells
Source: Front Immunol. 2017 Jan 23;8:7. doi: 10.3389/fimmu.2017.00007 (PMC5256039; doi:10.3389/fimmu.2017.00007)
Supplement: Supplementary file 4 [file Presentation_1.pdf]

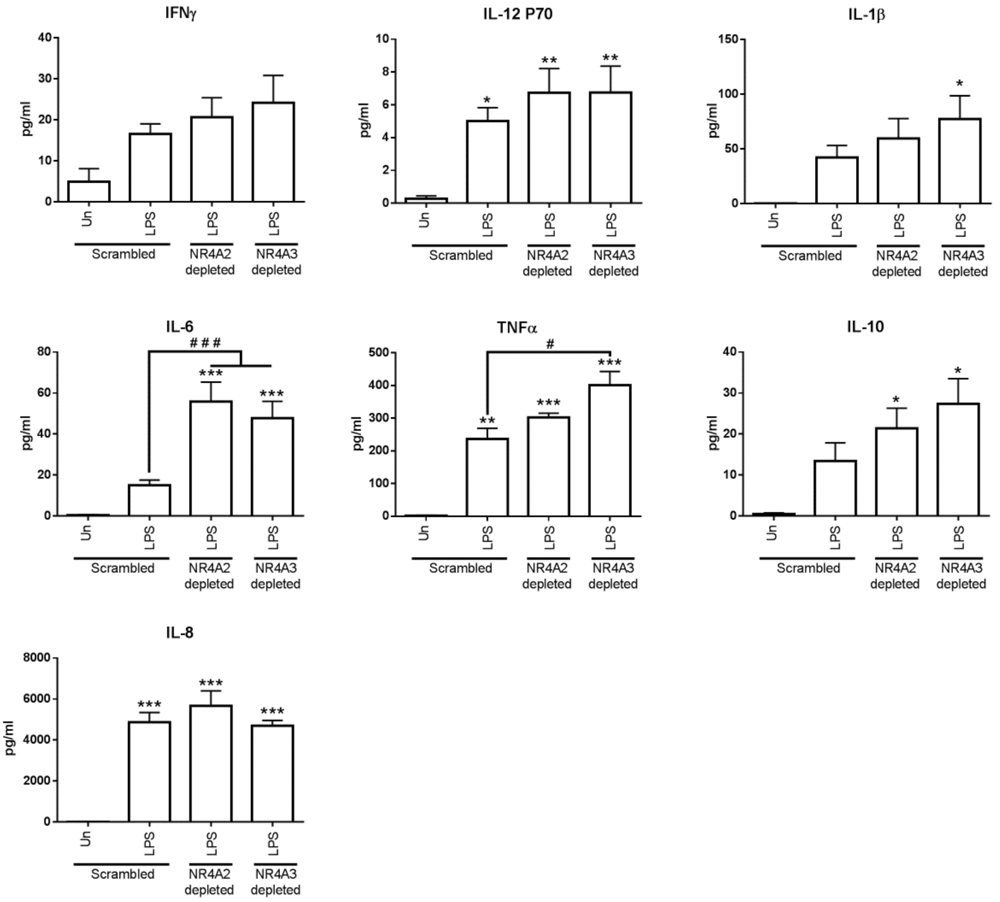

Supplemental Figure 1. NR4A receptors are important regulators of appropriate inflammatory responses. Undifferentiated THP-1 cells transduced with shRNA directed against scrambled non target control, NR4A2 or NR4A3 were treated with 50ng/ml LPS for 8hrs. Media was collected and human multiplex ELISA was performed to detect IFNγ, IL-12p70, IL-1β, IL-6, TNF, IL-10 and IL-8. Data are expressed as pg/ml ± SEM for n = minimum of 3 individual experiments. *p< 0.05, **p< 0.01, ***p< 0.001 treatments compared to untreated control (Un). $^{\#}$p< 0.05, $^{\#\#\#}$p< 0.001 treatments compared displayed here using a bar attachment.

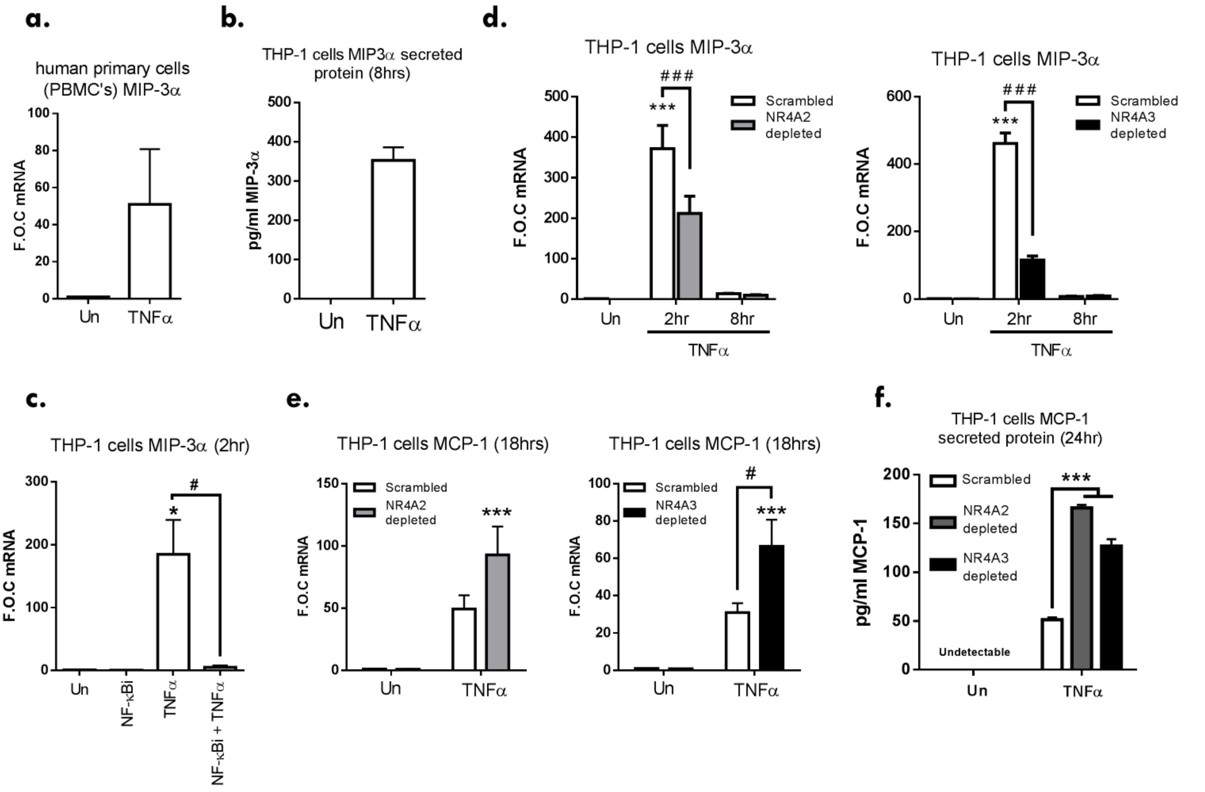

Supplemental Figure 2: NR4A2 and 3 regulate TNFα driven NF-κB target genes MIP-3α and MCP-1 in human monocytes. a,b) Human primary PBMC's and THP-1 cells were exposed to 5ng/ml TNFα for 2hrs (a) and 8hrs (b), followed by RNA isolation and media collection respectively. c.) Undifferentiated THP-1 cells were pre-treated with 10µM NF-κB inhibitor (NF-κBi) for 1hr followed by the addition of 5ng/ml TNFα for 2hrs. d.) Undifferentiated THP-1 cells transduced with shRNA directed against scrambled non target control, NR4A2 or NR4A3 were treated with 5ng/ml TNFα for 0, 2hrs and 8hrs. e.) Undifferentiated THP-1 cells transduced with shRNA directed against scrambled non target control, NR4A2 or NR4A3 were treated with 5ng/ml TNFα for 18hrs. f.) Undifferentiated THP-1 cells transduced with shRNA directed against scrambled non target control, NR4A2 or NR4A3 were treated with 5ng/ml TNFα for 24hrs followed by media collection. Analysis: RNA was isolated and RT-PCR was performed at indicated times to assess levels of MIP-3α (a,c,d), MCP-1 (e) and control gene GAPDH (a,c,d,e). ELISA analysis was performed for MIP-3α (b) and MCP-1 (f) in media collected at times indicated above (b,f). Un: Untreated control. Data are expressed as fold over untreated control (F.O.C) or pg/ml ± SEM for n = minimum of 3 individual experiments. *$p < 0.05$, ***$p < 0.001$ treatments compared to untreated control (Un). $^{\#}p < 0.05$, $^{\#\#\#}p < 0.001$ treatments compared displayed here using a bar attachment.

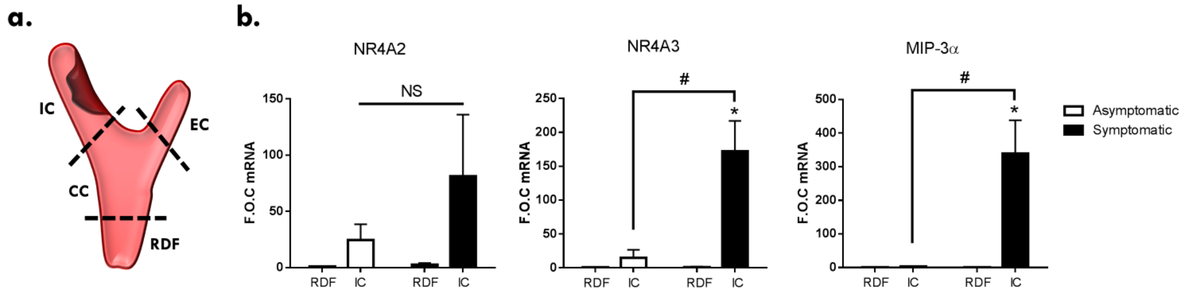

Supplemental figure 3: NR4A3 and MIP-3α expression significantly increased in symptomatic carotid atherosclerotic plaque. a.) Schematic of carotid atherosclerotic plaque dissection: The dotted lines indicate the points of dissection of the atherosclerotic plaque specimen, and the resulting sections are labelled as follows: Common Carotid (CC); the main arterial element and origin of disease, Internal Carotid (IC), the larger bifurcation from the CC; External Carotid (EC), the smaller bifurcation from the CC and the Relatively Disease Free component (RDF), least diseased region of the artery due to its peripheral location. b.) RNA was isolated from RDF and IC of asymptomatic (n=3) and symptomatic patients (n=3) and subsequent RT-PCR was performed to assess levels of NR4A2, NR4A3, MIP-3α and control gene 18S. Data are expressed as fold over asymptomatic RDF. NS = not significant. *$p < 0.05$ treatments compared to asymptomatic RDF. $^{\#}p < 0.05$ treatments compared displayed here using a bar attachment.

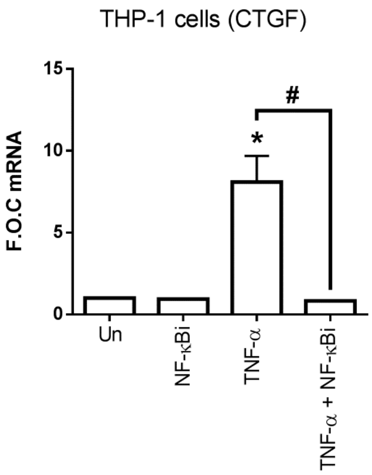

Supplemental Figure 4: NF-κB regulates TNFα driven MIP3α. Undifferentiated THP-1 cells were pre-treated with 10µM NF-κB inhibitor (NF-κBi) for 1hr followed by the addition of 5ng/ml TNFα for 2hrs. RNA was isolated and RT-PCR was performed to assess levels of CTGF and control gene GAPDH. Data are expressed as fold over untreated control (F.O.C) ± SEM for n = minimum of 3 individual experiments. *p< 0.05 treatments compared to untreated control (Un). #p< 0.05 treatments compared displayed here using a bar attachment.
